# The complete and fully assembled genome sequence of *Aeromonas salmonicida subsp. pectinolytica* and its comparative analysis with other *Aeromonas* species: investigation of the mobilome in environmental and pathogenic strains

**DOI:** 10.1186/s12864-017-4301-6

**Published:** 2018-01-05

**Authors:** Friedhelm Pfeiffer, Maria-Antonia Zamora-Lagos, Martin Blettinger, Assa Yeroslaviz, Andreas Dahl, Stephan Gruber, Bianca H. Habermann

**Affiliations:** 1 Computational Biology Group, Max Planck Institute of Biochemistry, Am Klopferspitz 18, 82152 Martinsried, Germany; 2 Research Group Chromosome Organization and Dynamics, Max Planck Institute of Biochemistry, Am Klopferspitz 18, 82152 Martinsried, Germany; 3 Biotechnology Center (biotec), Technical University Dresden, Tatzberg 47, 1307 Dresden, Germany; 4 Department of Fundamental Microbiology, University of Lausanne, 1015 Lausanne, Switzerland; 5 Computational Biology Group, Developmental Biology Institute of Marseille (IBDM) UMR 7288, Aix Marseille Universite, Parc Scientifique de Luminy, 163 Avenue de Luminy, 13009 Marseille, France

**Keywords:** *Aeromonas*, Complete circular genome, Transposon, Mobilome, PacBio, Long-read sequencing, High-precision sequencing, Mobilome-dependent evolution, Pathogenic, Environmental

## Abstract

**Background:**

Due to the predominant usage of short-read sequencing to date, most bacterial genome sequences reported in the last years remain at the draft level. This precludes certain types of analyses, such as the in-depth analysis of genome plasticity.

**Results:**

Here we report the finalized genome sequence of the environmental strain *Aeromonas salmonicida subsp. pectinolytica* 34mel, for which only a draft genome with 253 contigs is currently available. Successful completion of the transposon-rich genome critically depended on the PacBio long read sequencing technology. Using finalized genome sequences of *A. salmonicida* subsp*. pectinolytica* and other Aeromonads, we report the detailed analysis of the transposon composition of these bacterial species. Mobilome evolution is exemplified by a complex transposon, which has shifted from pathogenicity-related to environmental-related gene content in *A. salmonicida* subsp*. pectinolytica* 34mel.

**Conclusion:**

Obtaining the complete, circular genome of *A. salmonicida* subsp. *pectinolytica* allowed us to perform an in-depth analysis of its mobilome. We demonstrate the mobilome-dependent evolution of this strain’s genetic profile from pathogenic to environmental.

**Electronic supplementary material:**

The online version of this article (10.1186/s12864-017-4301-6) contains supplementary material, which is available to authorized users.

## Background

The genus *Aeromonas* belongs to the *γ-proteobacteria*. Aeromonads occur in virtually every environmental niche, though they seem to be predominantly found in aquatic environments. Most known Aeromonads show pathogenicity to vertebrates (mammals, fish, birds) or invertebrates (arthropods, molluscs). The best-studied members of *Aeromonas* are consequently pathogenic species or from aquatic environments [1, 2].

*A. salmonicida* comprises five different subspecies: s*almonicida, achtomogenes, masoucida* and *smithia*, all of them isolated from fish, as well as *pectinolytica*, the only subspecies isolated from the environment. The five subspecies form a tight phylogenetic cluster, confirming their joint classification as subspecies of *A. salmonicida* [3]. Phylogenetic analysis of *Aeromonas* strains based on multi-locus sequence typing showed that the first four subspecies were more closely related, indicating that *A. salmonicida* subsp*. pectinolytica* is the phylogenetically most distant member [4]. Several novel isolates of *A. salmonicida* were recently published, some of which may belong to a new subspecies (based on two isolates from India); another Indian isolate displays close similarity to *A. salmonicida* subsp*. pectinolytica* [5].

*A. salmonicida* subsp*. pectinolytica* strain 34mel was isolated from the water of a heavily polluted river that receives substantial industrial and urban sewage, including fuel hydrocarbons. This strain, which is resistant to various pollutants, produces abundant melanin [3]. A draft genome for this strain has been published [6] and an analysis of the genome has been described [7]. *A. salmonicida* subsp*. pectinolytica* is one of the few members of the *Aeromonas* genus that apparently lacks pathogenic potential. In contrast to *A. salmonicida* subsp. *salmonicida*, it is mesophile and grows well at 37 **°**C.

In a recent study, Vincent and colleagues sought to explain life-style evolution in *A. salmonicida* subspecies by comparative genomics [5]. Next to differences in pathogenic potential, the possibility to grow at higher temperatures is one discriminating feature of *A. salmonicida* subspecies (see Additional file 1: Table S1): most *A. salmonicida* subspecies are psychrophilic, i.e. they are cold-adapted and only grow at low temperatures. However, *A. salmonicida* subsp*. pectinolytica*, as well as the three newly sequenced Indian isolates are mesophilic (able to grow at higher but still moderate temperatures). *A. salmonicida* subsp*. masoucida,* initially defined as psychrophilic, is able to grow at 37 **°**C albeit slowly. The mobilome of *A. salmonicida* was identified as one of three functional categories that differ most between mesophilic and psychrophilic subspecies; it was subsequently hypothesized as one of the driving forces in mesophilic-to-psychrophilic transition. Their mobilome analysis involved transposons, which our group has identified in the 34mel strain and other completely sequenced *Aeromonas* genomes with subsequent submission to the ISFinder database in an early phase of the currently described project. However, a precise description of the species’ mobilome depends on a complete, circular genome.

Here we present the complete, fully assembled genome of *A. salmonicida* subsp*. pectinolytica* strain 34mel. We discuss our genome sequencing strategy, which is based on single-molecule real-time long read sequencing (PacBio), the high-quality annotation of the genome and a detailed and comparative analysis of the mobilome in different environmental and pathogenic *Aeromonas* species with a fully assembled genome.

## Results

### Genome sequencing strategy and verification of the *A. salmonicida* subsp. *pectinolytica* genome

Next-generation sequencing approaches such as 454-sequencing or Illumina-sequencing encounter severe difficulties when genomes contain a large number of long repeats. For *A. salmonicida* subsp*. pectinolytica* strain 34mel, there is currently only a draft genome available, which consists of 253 contigs [6]. We therefore set out to obtain a complete and final genome sequence of this strain. Our strategy involved three different sequencing technologies (Additional file 1: Fig. S1): single-molecule real-time sequencing (PacBio SMRT), as well as sequencing after clonal amplification (Illumina sequencing and Roche 454 pyrosequencing). Reads from PacBio, as well as from 454 sequencing were individually de novo assembled, while short reads from Illumina sequencing were used to verify the sequence of the resulting assemblies.

De novo assembling the 454 reads proved to be difficult, as we obtained a scaffold with 166 contigs plus two additional short contigs. Besides rRNA operons, the large number of transposons (see below) interfered with the 454 assembly. We closed gaps via generation and sequencing of PCR products. However, for several gaps the sequence was unobtainable even using standard Sanger sequencing. Thus, we prepared a one-contig version of the genome by ordering the contigs along the scaffold and insertion of poly-N stretches of the expected length at contig gaps. Genome sequencing using the PacBio long read sequencing technology, which has already proven as the superior technique for bacterial genomes compared to short-read sequencing [8], was more successful. We could de novo assemble the long PacBio reads into a single contig representing the full-length, circular genome.

We next compared the two assemblies using an in-house developed script for analysis of very closely related genomes [9]. In order to focus on the relevant regions of the 454 contigs, we replaced poly-N stretches at contig gaps with the sequences occurring at the corresponding positions in the PacBio assembly. Our analysis showed that the original 454 assembly represented nearly all of the unique sequences from the genome. Only 68 differences of one or few bases were detected between the assemblies in the unique regions.

We attempted to resolve these discrepancies and thus mapped Illumina reads to each assembly (Additional file 1: Fig. S1, for details see Methods). All of the 68 differences observed between the PacBio and 454 assembly could be resolved with the Illumina reads. In the majority of cases (60 positions), the Illumina sequences supported the PacBio assembly and thus disproved the 454 assembly. In the remaining 8 cases, the PacBio sequence had one base missing in a poly-C or poly-G region. Six of the 8 indels were within coding regions and a BLASTx analysis against UniProt suggested the necessity to correct the original PacBio sequence.

**Fig. 1:**
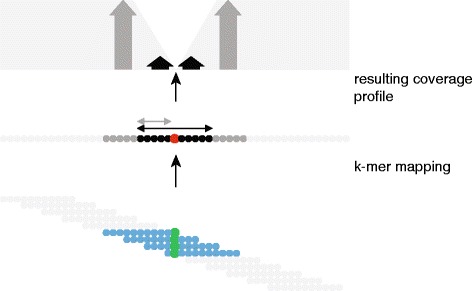
Illustration of the k-mer analysis procedure. For space reasons, the k-mer analysis is illustrated with 11-mers and 3-base step size (while 49-mers with one-base step size were actually used). The central line represents the genome sequence, each circle one base. The black arrow indicates one k-mer length (11 bases), the grey arrow a half-kmer ((kmer-1)/2, thus 5 bases); The central base (red) marks a divergent base pair, which represents in our case a sequencing error. Below are k-mers extracted from Illumina reads, which have the correct base (green) at the position of the sequencing error. K-mers, which do not contain the central base (indicated by grey color) are present in the genome and thus contribute to genome coverage. In our algorithm, the coverage count is increased at each of the bases covered by the k-mer. In contrast, k-mers, which contain the central base (indicated by blue color) are not present in the genome and thus do not contribute to genome coverage. Genome coverage is schematically illustrated in the top graphic (light grey shading). Starting left, genome coverage stays high as long as k-mers do not reach the central base and then drops continuously up to the central, incorrect base. Thereafter, coverage increases continuously and reaches full coverage as soon as the first k-mer base is beyond the central base. For computation of coverage drops, bases adjacent to the query base are classified as inner bases (black) and outer bases (dark grey); inner bases are a half k-mer on each side of the query base and outer bases are the adjacent half k-mer. The sum of coverage is computed over inner bases (illustrated by the black arrow) and also over outer bases (illustrated by the dark grey arrow). The coverage ratio of outer bases over inner bases is the coverage drop. High coverage drop values indicate potential genome sequencing errors. Frequently occurring read k-mers, which are not found in the genome (blue) may also hint to genome sequence problems

We became aware that the Illumina read mapping procedure was partially incomplete, as overly stringent read mapping parameters were initially used. Therefore, we repeated Illumina read mapping with relaxed parameters and detected additional differences in one of the rRNA operons (operon J). Additionally, we observed significant coverage drops in rRNA operon J and in some other parts of the genome. To further investigate these regions, we developed a validation algorithm based on k-mer analysis: we built one catalog of 49-mers from the genome, and one from the Illumina reads, respectively and compared those against each other. This allowed us to identify frequently occurring read k-mers, which were not represented in the genome sequence, and to compute the k-mer coverage at each genome position. Then, ***coverage drops*** were computed, which are expected to be high if a divergent base, which could represent a sequencing error or a true point mutation, is encountered (Fig. 1, see Methods for full details). We also computed ***coverage slopes***, which are expected to be high in case of a larger indel. Applying this procedure, we detected further differences in rRNA operon J. Altogether, 38 differences over a region of ~3 kb were detected in that operon. We further corrected this part to result in a final genome sequence, which was used for all further analyses. We have successfully applied the same procedure to identify sequencing errors in another bacterial genome [10].

We were astonished to find 38 differences over 3 kb in rRNA operon J, while the remainder of the genome had only 8 sequencing errors. A possible explanation is the extreme similarity of rRNA operon J with operons H and C. There are only 13 and 15 differences from the start of the 16S rRNA to the end of the 23S rRNA, respectively, with only two point mutations in the 16S/23S linker region. Thus, many reads originating from rRNA operon J may have been assigned to operons C or H by the assembler, leaving only a low coverage of predominantly diverging reads for operon J.

### Genome sequence and gene content of *A. salmonicida* subsp. *pectinolytica* strain 34mel

The genome of *A. salmonicida* subsp*. pectinolytica* consists of a single chromosome of 5,012,649 base pairs (bp) with 58.3% GC content (Additional file 1: Table S2). The position to open the circular genome was chosen according to the *A. salmonicida* subsp*. salmonicida* strain A449 genome upstream of *dnaA*. There are 10 rRNA operons, showing a number of polymorphisms (Additional file 1: Table S3). These rRNA operons can be considered large-scale duplications and are a challenge for genome finishing. The correct assembly across these rRNA operons illustrates the efficiency of the PacBio long-read approach. We encountered 370 PacBio reads, which completely traverse the rRNA operons, of which 369 support our genome assembly (see Methods for details). Each of the rRNA operons is supported by at least 24 distinct PacBio reads.

**Fig. 2:**
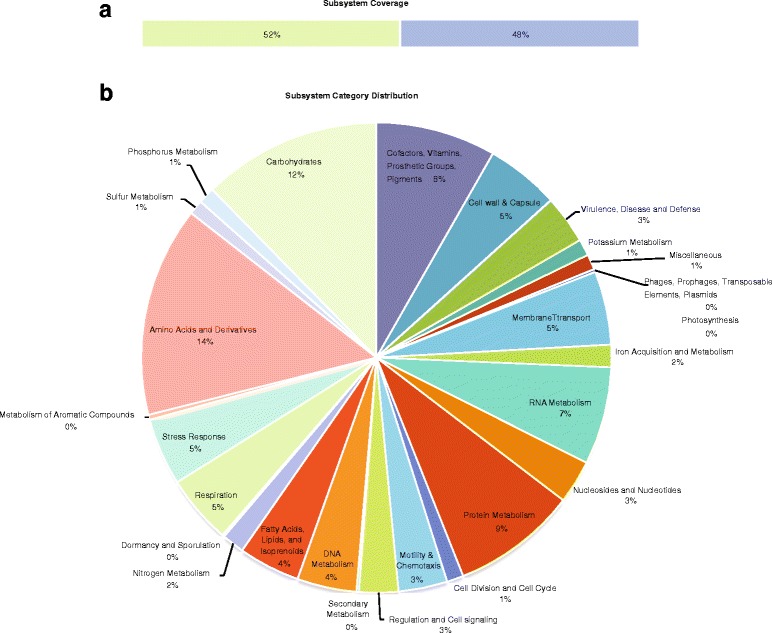
RAST annotation summary. After gene calling, the RAST annotation robot assigns names and functions to protein-coding genes via their subsystem technology. The overall subsystem coverage (**a**) and the breakdown into various function classes (**b**) are illustrated

Genome annotation was done using the following strategy: first, we ran a genome annotation using the RAST server [11]. This resulted in the annotation of 4502 protein-coding genes and 155 RNAs. RAST uses so-called subsystems, which are manually curated sets of abstract functional roles that typically describe and unite genes, which are part of a specific pathway. According to the RAST classification, the genome contains 530 subsystems, covering 2334 (52%) of the protein-coding genes (Fig. 2). In the second step, we searched for missing gene calls, evaluated the start codon assignments and manually corrected those that were inconsistent with homologs. Finally, we enhanced the annotation of disrupted genes (pseudogenes) to collect all segments represented in the genome, also removing invalid sequence extensions.

We decided to manually curate the automatic annotation following the general annotation strategy of halophilic archaea [12] and for *Halomonas elongata* [10]. For protein-coding genes with close-enough homologs in the SwissProt section of UniProt, annotation differences were reconciled. Proteins with specific function assignments lacking close homologs in SwissProt were further evaluated. In a few cases, we could identify a functionally characterized homologous protein by literature search. In such cases, the published characterization data are not available via SwissProt and we requested to update this missing information in the database. In the majority of cases we could not identify a functionally characterized homolog. Consequently, we considered the specific function assignment an over-annotation error and revised the annotation to only a general protein name. After manual curation, the genome contains 4590 protein-coding genes, 209 of which are disrupted genes (Additional file 1: Table S4). We also improved the annotation of RNA genes, which resulted in a total of 165 RNAs (for details, see Methods and Additional file 1: Text S1).

### Comparison to the *A. salmonicida* subsp. *pectinolytica* strain 34mel draft genome sequence

Even though the draft genome of strain 34mel consists of 253 contigs, it represents all of the unique sequences of our finished genome. Most of the contigs representing unique genome regions terminate at rRNA operons or transposons. A considerable number of the contigs are (partial) transposons or internal rRNA operon segments. In general, the genome sequences agree quite well. However, there are a number of differences (point mutations or short indels). Only three short contigs from the draft genome are not present in the complete genome sequence (see Additional file 1: Text S2).

### Comparative analysis of gene content to other *Aeromonas* species with a complete genome sequence

**Fig. 3:**
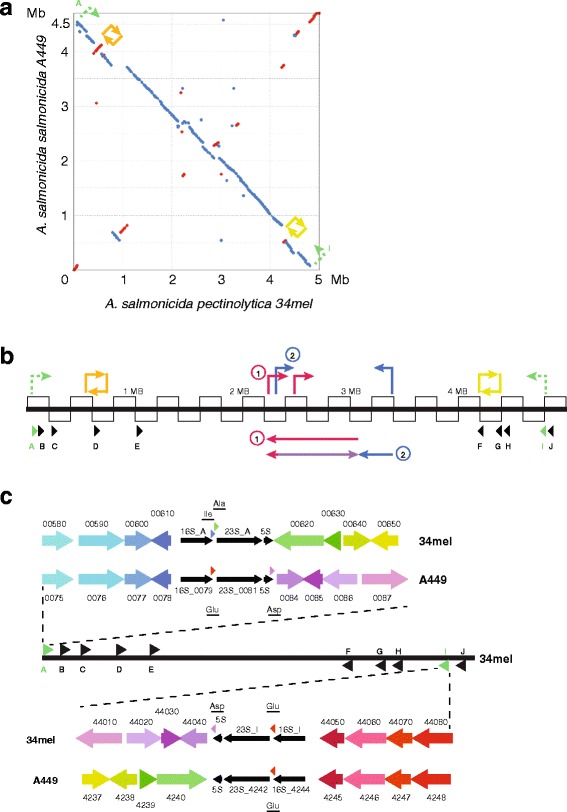
Comparison of the genomes from *A. salmonicida* subsp*. pectinolytica* strain 34mel and from *A. salmonicida* subsp*. salmonicida* strain A449. **a** Mummer-based genome alignment. Red dots/lines indicate matches in forward orientation, blue dots/lines matches in reverse orientation. Colored arrows indicate genome inversions. **b** Schematic representation of the 5 Mb genome from strain 34mel (thick line) with each black box representing a 200 kb region. Small arrowheads with uppercase letters represent the 10 rRNA operons. Those responsible for the 4 Mb inversion (A and I) are indicated by green coloring and dashed bent arrows. Four transposon-triggered inversions are indicated by bent arrows (small inversions not drawn to scale, further details in Fig. 4a-d. Two serial overlapping inversions are indicated. The first inversion (labeled “1”, indicated by red arrows and by the long red line with an arrowhead indicating reverse orientation) was partially re-inverted by a subsequent inversion (labeled “2”, indicated by blue arrows). The re-inverted section is represented by the purple long line, forward orientation is indicated by the arrowhead. **c** Schematic representation of rRNA operons A and I with the adjacent four protein-coding genes in strains 34mel and A449. Identical color indicates highly conserved sequences. The inverse relationship of the inner genes is obvious

There are four *Aeromonas* strains with a fully sequenced, circular genome in the RAST system. All of those are identified as closely related genomes of our strain 34mel with the following percentages of proteins with more than 90% sequence identity: *A. salmonicida* subsp. *salmonicida* strain A449 (77.0%), *A. hydrophila* subsp. *hydrophila* ATCC 7966 (58.9%), *A. media* strain WS (48.4%) and *A. veronii* strain B565 (46.0%) (see Additional file 1: Fig. S2 and Fig. 3a for protein similarity overview and for Mummer-based genome alignments [13]). *A. hydrophila* is human-pathogenic, as is *A. veronii* [14], strains of which are symbionts to leeches [15]. *A. media* strain WS, on the other hand, was isolated from East Lake (Wuhan, China), exhibiting high yield of melanin [16] and can be considered an environmental strain.

*A. salmonicida* subsp*. salmonicida* strain A449 [17] is one of the best-characterized closely related genomes to *A. salmonicida* subsp*. pectinolytica* strain 34mel. The two genomes align primarily in reverse orientation (Fig. 3a). Many of the matching regions fall along the diagonal, indicating a high level of gene synteny. The X-shaped patterns in such whole-genome alignments are considered to result from chromosomal inversions centered around the replication origin [18]. The opposite orientation of strains 34mel and A449 can be attributed to a 4.7 Mb genome inversion in the first and second-last rRNA operon of strain 34mel (Fig. 3a-c). On each side, at least the four adjacent genes are well conserved. While the set of outer genes is in parallel orientation, the set of inner genes is in opposite orientation (Fig. 3c).

### Comparative analysis of the mobilome to other *Aeromonas* species with a complete genome sequence

We performed extensive analysis of transposons in the *A. salmonicida* subsp. *pectinolytica* strain 34mel genome, as well as the other *Aeromonas* species with a complete genome. *A. salmonicida* subsp*. pectinolytica* carries a plethora of transposons of various types. A total of 218 transposons and transposon fragments were identified (Table 1 and Additional file 1: Table S5). Some transposon types are numerous, such as IS5 (37 copies), ISAs1, ISAs24, ISAs27, ISAs30, ISAhy2 (10–16 copies).

**Table 1:** Transposon summary

Strain tag	Transposon types	Distinct transposons (all)	Distinct transposons (considering complete copies)	Number of transposon copies (all)	Number of complete transposon copies
*A. media*	32	76	52	370	308
*A. s. pectinolytica*	30	56	36	218	173
*A. s. salmonicida*	21	51	20	143	95
*A. veronii*	12	16	7	30	17
*A. hydrophila* AL06	8	16	8	25	17
*A. hydrophila* 7966	4	8	1	9	2

Transposons have been systematically analyzed in six *Aeromonas* strains with a final complete sequence. We report the total number of copies including fragments (all) as well as complete copies. Related transposons are grouped into the same transposon “type” and the number of distinct transposon types (considering all transposons) is also reported. Strains are: *A. s. pectinolytica* (*A. salmonicida* subsp*. pectinolytica* strain 34mel), *A. s. salmonicida* (*A. salmonicida* subsp*. salmonicida* strain A449), *A. media* (*A. media* strain WS), *A. veronii* (*A. veronii* strain B565), *A. hydrophila 7966* (*A. hydrophila* subsp. *hydrophila* ATCC 7966, type strain), *A. hydrophila* AL06 (*A. hydrophila* subsp. *hydrophila* strain AL06–06)

Transposons frequently target each other, leading to repeat conglomerates. As an example, ISAs19_PB has been targeted twice: once by ISAhy2; and once by ISAs27, which was in turn targeted by ISUnCu16. The complete conglomerate is 6.5 kb in size (see Additional file 1: Fig. S3). Several PacBio reads traverse this repeat conglomerate and confirm the assembly, which would typically result in a major challenge for genome assembly using shorter reads. In fact, all repeat conglomerates exceeding 4 kb have been verified by PacBio reads.

ISFinder considers all sequences with at least 95% sequence identity isoforms of the same transposon, even if they occur in distinct species (see Additional file 1: Text S3 for further details). By this convention, *A. salmonicida* subsp*. pectinolytica* carries several transposons, which are assigned to enterobacteria (IS5, ISKpn3, ISKpn10), to other species from the genus *Aeromonas* (ISAhy2, ISApu1, ISApu2, ISAeca1), or are of uncertain origin (ISUnCu16). All these transposons also occur in *A. media* strain WS*,* a mesophilic *Aeromonas* strain isolated from the environment, but not necessarily in other *Aeromonas* species (see Additional file 1: Table S5).

Transposons with reduced sequence identity classify to be distinct transposons in ISFinder. Among these newly identified transposons, several are related to elements initially detected in enterobacteria: IS2 (ISAs17), IS4 (ISAs30), IS903 (ISAs13, ISAs14, and ISAs15), ISEc12 (ISAs27, ISAs28, ISAs29), ISKpn10 (ISAs22), ISKpn15 (ISAs21), ISSen1 (ISAs31). Only one of the newly identified transposons is related to a pre-existing *Aeromonas* transposon (ISAs23 to ISAhy1). Some are related to other transposons (ISAs20 to IS1240, ISAs16 and ISAs24 to IS1328, ISAs26 to IS1341, ISAs19 to IS1419, and ISAs25 to ISShe12). Transposon ISAs18 is completely new without any ISFinder homolog on the DNA sequence level, but with 49% sequence identity to the *Anaeromyxobacter* transposon ISAnsp7 on the protein sequence level.

In addition to strain 34mel, we also performed an exhaustive transposon analysis of the *Aeromonas* species, for which a complete genome sequence is available, including *A. salmonicida* subsp*. salmonicida* strain A449, *A. hydrophila* (2 of the 10 available genomes), *A. veronii*, and *A. media* (Table 1, Additional file 1: Table S5). In total, 53 transposons were newly defined during our analysis and have been submitted to and accepted by the ISFinder database [19] (Additional file 1: Table S5). Of these 53 transposons, almost half (21) were from strain 34mel. One transposon, ISAs29 from the IS21 family, closely matches the characteristics, which are listed for ISAs11 in Table 2 in Reith et al. [17]. However, the name ISAs11 has been reassigned as a synonym for the IS256 family transposon ISAs3 (for more details, see Additional file 1: Text S4). We also identified and submitted three MITEs (Miniature Inverted-Terminal-repeat Elements).

We observed a difference in transposon composition, and also in counts between the environmental and the pathogenic strains (Additional file 1: Table S5 and Additional file 1: Fig. S4). While the human pathogenic *A. hydrophila* and *A. veronii* carry only a small number of transposons or fragments thereof (9–30 copies, of which 2–17 are complete), *A. salmonicida* subsp*. salmonicida* strain A449 harbors significantly more (143 copies, 95 complete). The genomes from non-pathogenic, environmental isolates show the highest numbers of transposons (218/173 in *A. salmonicida* subsp*. pectinolytica* strain 34mel, 370/308 in *A. media* WS). When we compared the mobilomes on the level of IS families, we found more similarity between *A. salmonicida* subsp. *pectinolytica* 34mel and *A. media* WS than between the two *A. salmonicida* subspecies (Additional file 1: Fig. S4). 19 transposons are shared between *A. salmonicida* subsp*. pectinolytica* strain 34mel and *A. media* strain WS, only 5 of which are also found in *A. salmonicida* subsp*. salmonicida* strain A449. All elements that are shared between strains 34mel and A449 can also be found in *A. media* strain WS, when the analysis is restricted to complete transposons. There are many transposons specific for only one of these genomes (11 in strain 34mel, 8 in strain A449, 28 in *A. media*, when the analysis is restricted to complete transposons).

**Fig. 4:**
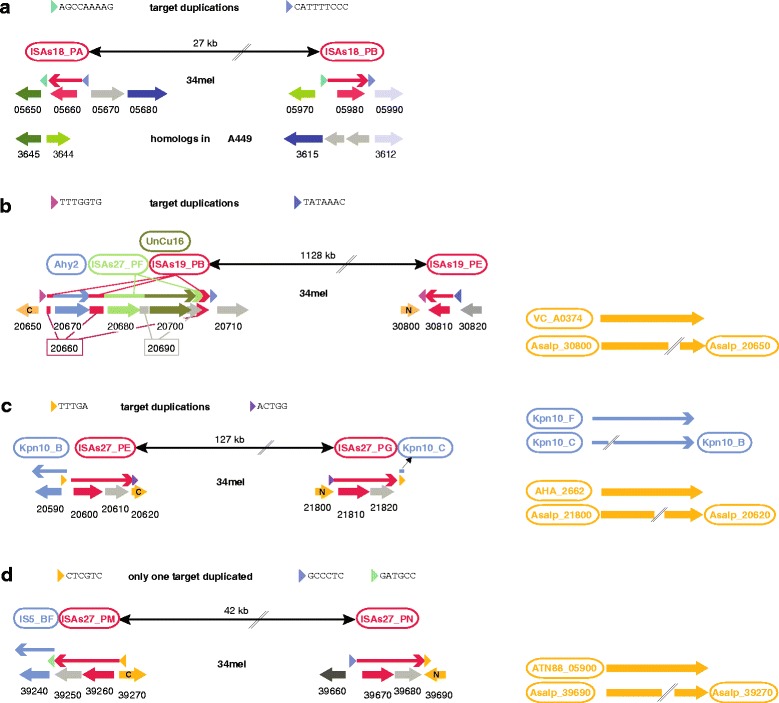
Transposon-triggered inversions. In each panel, the second row indicates the pair of transposon copies, which triggered the inversion, and their genomic distance. Thin arrows in the 3^rd^ row indicate transposons with target duplications drawn as colored triangles. The corresponding target duplication sequences are shown in the 1^st^ row. In the 4^th^ row, protein-coding genes are shown by thick filled arrows with an indication of the integer part of the ordered locus tag (the term “Asalp_” was omitted for space reasons). For protein-coding genes, which are split into two fragments, the N-terminal fragment is labeled “N”, the C-terminal labeled “C” (see also Additional file 1: Table S4). Additional illustrations, which lend further support for the interpretation as a genome inversion, are panel-specific. **a** The adjacent protein-coding genes and their genomic clustering are illustrated for *A. salmonicida* subsp*. salmonicida* strain A449 with highly conserved sequences specified by identical coloring. **b** One of the transposon copies (ISAs19_PB) belongs to a complex transposon conglomerate as indicated by additional transposons in the 2^nd^ row (see also Additional file 1: Fig. S3 B). This transposon has targeted a protein-coding gene, which, after inversion, is split into two genomically distant fragments (Asalp_30800 and Asalp_20650). These two fragments together are the full-length homolog of VC_A0374 from *Vibrio cholerae* with 51% sequence identity. **c** One transposon copy of ISAs27 has targeted another transposon (Aersa_Kpn10), which is split into two fragments as indicated in the 2^nd^ row. The two fragments combine to a single complete element with just an internal insertion of one target duplication. The other transposon copy of ISAs27 has targeted a protein-coding gene, which, after inversion, is split into two genomically distant fragments (Asalp_21800 and Asalp_20620). These fragments are together a full-length homolog of AHA_2662 from *Aeromonas hydrophila* with 95% sequence identity. **d** One of the transposon copies has targeted a protein-coding gene, which, after inversion, is split into two genomically distant fragments (Asalp_39690 and Asalp_39270). These fragments are together a full-length homolog of ATN88_05900 from *Enterovibrio coralii* with 44% sequence identity. One of the elements has targeted a transposon (Aersa_IS5), which is incomplete. The targeting element was probably affected by a subsequent rearrangement so that the residual part of the targeted transposon is absent and a 2^nd^ target duplication is not encountered

In the strain 34mel genome, we detected several genome inversions while analyzing target duplications, which commonly surround transposons. In such cases, target duplications are reciprocal, preceding one copy of a transposon but following another copy. These inversions were strongly supported by genome context analysis (Fig. 4a-d):

(a) A 27 kb genome inversion between strain 34mel and strain A449 is bounded by transposon ISAs18 copies PA and PB, which show a reciprocal 9 bp target duplication (Fig. 4a). In the strain A449 genome, the genes downstream of ISAs18_PA and upstream of ISAs18_PB are adjacent. Similarly, the 2^nd^ gene upstream of ISAs18_PA and the gene downstream of ISAs18_PB are in close proximity in the strain A449 genome.

(b) A reciprocal 7 bp target duplication was found between ISAs19 copies PB and PE, which are 1.13 Mb apart (Fig. 4b). As described above, ISAs19_PB has been targeted twice (Additional file 1: Fig. S3 B). The sequence downstream of ISAs19_PE and the sequence upstream of ISAs19_PB code for the N-terminal and C-terminal parts of a pseudogene, which together show 51% sequence identity to VC_0374A from *Vibrio*.

(c) The ISAs27 copies PE and PG, which are 127 kb apart, show a reciprocal 5 bp target duplication (Fig. 4c). The partial Aersa_Kpn10_B upstream of ISAs27_PE and the partial Aersa_Kpn10_C downstream of ISAs27_PG combine to a perfect complete element with an insert of just a 5 bp target duplication. The sequence upstream of ISAs27_PG and the sequence downstream of ISAs27_PE code for the N-terminal and C-terminal parts of a pseudogene, which together are a close homolog of AHA_2662 from *A. hydrophila*. Unexpectedly, both elements are on the same strand. However, ISAs27_PG is located on the 1.13 Mb inversion (see above, b), only 2 kb from its left junction. It thus can be assumed that inversion c was originally a 1.1 Mb inversion, terminating 2 kb upstream of the right junction of inversion b, and that 1.0 Mb of this has been reverted back to the original orientation during inversion c (as illustrated in Fig. 3b). Accordingly, this pair of inversions does not show up prominently on the Mummer alignment of the two genomes (Fig. 3a).

(d) The ISAs27 copies PM and PN, which are 42 kb apart, share one 6 bp target duplication (Fig. 4d). The sequence downstream of ISAs27_PN and the sequence upstream of ISAs27_PM code for the N-terminal and C-terminal parts of a pseudogene, which together are a close homolog of ATN88_05900 from *Enterovibrio coralii*. The partial transposon Aersa_IS5_BF downstream of ISAs27_PM does not have a counterpart upstream of ISAs27_PN. This implies that these regions are no longer original due to subsequent rearrangements. Accordingly, the immediately adjacent sequences are distinct and do not represent a 2^nd^ target duplication.

**Fig. 5:**
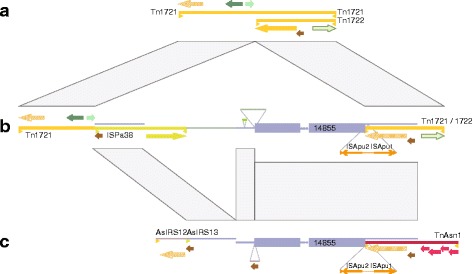
Comparison of the strain 34mel genome, plasmid pFBAOT6 from *Aeromonas caviae*, and transposon Tn1721. The three “panels” represent **a** transposon Tn1721, **b** a segment of plasmid pFBAOT6 (pos 83,802–41,003) and **c** a segment of the strain 34mel genome (pos 4,961,312–4,993,921). The shared segments are connected by gray boxes. In all panels, filled thick arrows indicate regular protein-coding genes, “striped” arrows indicate pseudogenes. Coloring: yellow colors: transposase; brown: resolvase; green colors: tetracycline resistance genes; light-green with dark-green border: methyl-accepting chemotactic protein; red: TOL plasmid related genes. For reasons of clarity, many protein-coding genes are not shown (see Additional file 1: Fig. S5 for more detailed images). **a** The top panel shows extended transposon Tn1721 and the basic transposon Tn1722 (yellow lines). Inverted terminal repeats are indicated by yellow triangles (not drawn to scale). **b** The middle panel shows the region from plasmid pFBAOT6 where Tn1721 is interrupted by two insertions. The part of Tn1721, which is also found in the 34mel genome is indicated by the thin blue line right above the yellow line of Tn1721/1722. A 3 kb insertion, which represents a transposon cassette, is drawn as an inserted line: thin orange arrows indicate ISApu1 and ISApu2 positioned in opposite orientation. The target duplication, which encloses the complete cassette, is indicated by orange triangles (not drawn to scale). The central thin line represents a 642 bp segment, which is carried along on the transposon cassette. A 28 kb insertion is drawn “in-line”. This region terminates with a copy of the Tn3-related transposon ISPa38 (drawn in yellow-green with terminal inverted repeats indicated by yellow-green triangles). The region, which is also found in the 34mel genome is indicated by the blue line drawn right above ISPa38 and its transfer is also indicated. A sequence, which is unique to pFBAOT6 is indicated by a thin green line. Three segments, which are shared between pFBAOT6 and the 34mel genome are indicated by two thin blue lines and an interrupted box (not drawn to scale, total length 14855 bp). The 206 bp MITE MITEAeca1, which is found in pFBAOT6 but not in the 34mel genome, is placed above the main sequence line and highlighted in bright green. An 1865 bp sequence, which is unique to pFBAOT6 and is replaced by an iso-positioned resolvase gene in the 34mel genome is drawn in green above the main sequence line. **c** Transposon TnAs1 is drawn as a dark-red thick line. The element AsIRS12, which terminates with an inverted terminal repeat (yellow triangle) is indicated by a thick grey line. For all other markup see panel (**b**)

Transposons TnAs1 and TnAs2 in strain 34mel and TnAs3 in strain A449 belong to the ISFinder Tn3 family. Elements named “Tn” carry a resolvase in addition to the transposase gene. Tn3-like transposons in general are known to play a role in the spread of pathogenicity determinants [20]. These elements can carry large numbers of passenger genes because of a 38 bp long, perfect inverted terminal repeat, which allows for large transposon sizes (>10 kb). An example is Tn5393, which carries four distinct antibiotic resistance genes. These contribute strongly to the multi-resistance of *Aeromonas sp.* ARM81 [21]. Another characterized member is Tn1721 [22], which carries three copies of an inverted terminal repeat. It consists of a basic transposon of 5.4 kb (named Tn1722), which is enclosed by left and right inverted terminal repeats. Tn1721 extends beyond the right terminal repeat for another 5.3 kb, carrying tetracycline resistance genes, a partial highly conserved copy of the transposase gene from Tn1722, and a second right terminal repeat (Fig. 5, Additional file 1: Fig. S5 E).

In the genome of strain 34mel, we found a variant of Tn1722, which was submitted to ISFinder as TnAs1 (Fig. 5, Additional file 1: Fig. S5 B). It is chimeric, being near-identical over half of the transposon and completely unrelated in the other half. The common region encodes the transposase and resolvase, but with a 3415 bp insertion close to the C-terminus of the transposase gene in TnAs1. The unrelated parts of Tn1721 and TnAs1 encode unrelated genes: Tn1722 codes for a methyl-accepting chemotactic protein (MCP), which has been shown to interfere with chemotaxis upon overexpression [22]. The TnAs1-specific sequence is near-identical to a region from the IncP-9 TOL plasmid pWW0 from *Pseudomonas putida* (Fig. 5, Additional file 1: Fig. S5 B). Four genes were retained from this environmental plasmid (Additional file 1: Table S6): two enzymatic proteins, one of which contains an osmC domain, a conserved domain that is also found in proteins responsible for organic hyperoxide detoxification [23]; and two putative transcriptional regulators. One of these is closely related to the anti-sigma factor ChrR, which regulates gene expression in response to stress signals [24]. Tn1722 is the basic transposon of Tn1721, which has an extension on the side that is retained in TnAs1. We did not find the tetracycline resistance genes encoded within the Tn1721-specific extension in the 34mel genome. Overall, this exemplifies a shift in *A. salmonicida* subsp. *pectinolytica* from pathogenicity-related to environmental-related genes.

We next investigated the genetic origin of the chimeric transposon TnAs1. The common part of TnAs1 and Tn1722 belongs to a large region, which must have been acquired by horizontal gene transfer. This region originates from the 85 kb plasmid pFBAOT6; or both, TnAs1 and pFBAOT6, have obtained it from the same ancestral source. The 34mel genome shares 26,606 bp of complete sequence identity with plasmid pFBAOT6 in 4 sections, the longest having 22,017 bp (Fig. 5, Additional file 1: Fig. S5 A-D). Total sequence identity over such a long region is a strong indicator of a very recent acquisition of foreign genetic material. Plasmid pFBAOT6, which has been isolated from *A. caviae*, has a complete copy of Tn1721 with two insertions of 3415 bp and of 28,247 bp (Additional file 1: Fig. S5 CD). The common region between the 34mel genome and plasmid pFBAOT6 covers the mobilome-related part of the basic transposon Tn1722, all of the 3.4 kb insertion, which represents a transposon cassette, and long regions at the ends of the 28 kb insert. As mentioned above, the 34mel genome lacks the Tn1721-specific extension, which immediately follows the 28 kb insert. Instead, the 34mel genome contains an element, named AsIRS13, which has high similarity to the Tn1721-specific transposase pseudogene region and terminates with a 3^rd^ copy of the inverted terminal repeat. Thus, the overall structure of Tn1721 is retained in the 34mel genome. Because pFBAOT6 is near-identical to Tn1721, while the 34mel genome has rearrangements at both termini, the genetic flow must have been from Tn1721 to pFBAOT6 and then to the 34mel genome. A detailed comparison of Tn1721, pFBAOT6, and the region in the 34mel genome is provided as Additional file 1: Text S5.

## Discussion

We present here the complete, circularized genome sequence of *Aeromonas salmonicidae* subsp. *pectinolytica*, which we have used to perform a comprehensive comparative analysis of its mobilome to other *Aeromonas* species with a complete, circularized genome sequence.

Generally speaking, finishing the sequencing of a genome to its complete, circular form is a major challenge for genome projects, if a large number of repeats is encountered [8, 25]. It has become popular to skip this tedious phase and to release incomplete genomes in the form of a “permanent high-quality draft”. Even though this approach has its benefits, as the majority of the genome becomes accessible with limited effort, certain types of analyses become impossible due to the draft nature of the genome sequence. Here, we provide another example, which shows that the third generation PacBio SMRT sequencing technology with its exceedingly long read length re-opens the road to complete genome sequences. We provide a one-contig finished circular genome sequence for an organism, from which until now only a draft genome of 253 contigs has been available. The genome with its 10 rRNA operons and hundreds of transposons turned out to be an extreme challenge to first and second generation sequencing technologies. The quality of PacBio reads is sufficient to produce a trustworthy genome sequence without the need for further DNA sequencing: while long-read sequencing using PacBio initially showed very high error rates (>15%, [26]), more recent developments with this technology deliver highly accurate sequencing results given high enough coverage [27]. A clear benefit of the long-read PacBio SMRT technology is its ability to produce continuous long reads irrespective of sequence content. For instance, many long reads traverse highly repetitive regions in our bacterial genome without difficulties, making a complete, circular assembly of this genome possible.

The availability of a complete finished genome allowed us to carry out an extensive mobilome analysis. This analysis was not restricted to the newly generated complete genome of *Aeromonas salmonicida* subsp. *pectinolytica*, but was further extended to other complete genomes from the *Aeromonas* genus, the availability of which can in part be attributed to the major efforts taken by the sequencing groups to finish their genome. It is evident that the multiplicity of transposons varies dramatically between different strains of *Aeromonas*. Some carry only few transposons while others carry a plethora of such elements. Our analysis shows that pathogenic strains of *Aeromonas* typically carry only few transposons while environmental strains are studded with such elements. In order to make our knowledge available to the scientific community, we have submitted all the transposons and other mobile genetic elements, which we have identified during our studies to the ISFinder database [19].

The positive impact of such a submission can be seen by the analysis of Vincent et al. [5], who compared the mobilome of various draft genomes of *Aeromonas* strains. Having access to the transposons in ISFinder, many of which have been submitted by us in the course of the current project, they identified the mobilome as one of the key components, which differs between psychrophilic and mesophilic strains. We have focused our analysis on all complete *Aeromonas* genome sequences, which are available for a fish-pathogenic psychropilic strain, as well as several mesophilic strains that are environmental or human-pathogenic. The mesophilic human pathogens *A. hydrophila* and *A. veronii* have the lowest diversity and lowest number of mobilome elements, while the mesophilic environmental strains *A. media* and *A. salmonicida* subsp. *pectinolytica* have the highest diversity and number of mobile elements (Table 1). The two environmental strains also share unexpected similarity to each other with respect to the IS families they contain (Additional file 1: Fig. S4). It could be speculated that it is the distinction between the pathogenic and environmental nature of the strains, which dominates the large mobilome difference. Pathogenic strains are locked in a relatively narrow ecological niche and even a minor growth disadvantage attributable to the mobilome burden may result in an overgrowth by less affected members of the population. This may result in “genomic streamlining”. This hypothesis would be in accordance with reports from [28–30], which attribute a loss of virulence to genetic rearrangements of *A. salmonicida* subsp. *salmonicida* at temperatures higher than 22 °C. In contrast, environmental strains may explore a rich collection of ecological niches but also have a higher chance to get in contact with foreign DNA. Such foreign DNA, when internalized and integrated into the genome, may provide cells with enhanced metabolic capabilities at the expense of a more intense attack by selfish mobilome sequences. The integration of the TnAs1 transposon with its metabolic gene content may represent such an event in the 34mel genome. Upon a subsequent adaptation to the newly occupied ecological niche, replication efficiency may become again dominating so that genome, and especially mobilome reduction may regain a beneficial evolutionary impact. Yet, to address this question properly, the complete genome sequences of more closely related species from different niches (host versus environmental) and harboring different growth features (mesophilic versus psychrophilic) would be required.

## Conclusions

We have used PacBio long-read sequencing to obtain a finalized circular genome sequence of *Aeromonas salmonicida* subsp. *pectinolytica*. We have achieved high sequence reliability by comparing the final sequence assembly to Illumina short-read sequencing data using published mapping and variant calling algorithms, as well as a newly developed k-mer based mapping algorithm for detecting sequence discrepancies. We performed a detailed comparative analysis of the mobilome of *Aeromonas* species with a complete, circular genome and identified mobilome-dependent differences in gene content between environmental and pathogenic strains.

## Methods

### Genome sequencing and assembly

The genome sequence is primarily based on PacBio SMRT sequencing with 110-fold coverage (raw sequences: 642 Mb, 87215 reads with a mean length of 7358 bp, see Additional file 1: Text S6 for more details). In addition, 454 sequencing data (see below) and Illumina sequencing data (90 bp read length, 4166667 reads) were obtained. After trimming of Illumina raw reads, 666 Mb of reads were obtained, which corresponded to a 133-fold genome coverage. The PacBio sequences were assembled using the HGAP assembler (RS_HGAP_assembly.2) [31], which resulted in two contigs: one represented an untrimmed version of the full genome of *Aeromonas salmonicida* subsp. *pectinolytica*, with a size of 5036397 bp and a 110-fold mean coverage. The second, poorly covered contig of 6568 bp in size turned out to be a duplicated version of part of the genome sequence with considerable sequence variation (not considered further).

The 34mel genome sequence was trimmed to reflect the circularity of the genome and the position to open the circular genome was shifted to mirror that of the *A. salmonicida* subsp. *salmonicida* strain A449 genome. The resulting contig represented a draft version of the genome.

The validity of the assembly over each of the 10 rRNA operons was verified by identification of PacBio reads, which completely traverse each of them. Unique sequences (350 bp) on either side of each operon were used for BLASTn against the set of PacBio reads. A total of 370 PacBio reads traversed rRNA operons completely, having matches to upstream as well as downstream unique sequences (as revealed by a custom PERL script). All but one of these reads supported the PacBio assembly (as revealed by unique region correlation analysis). The correctness of the assembly over transposon conglomerates exceeding 4 kb was validated by the same method.

The 454 sequences and a subset of the Illumina sequences were co-assembled using the Newbler assembler (v 2.6) [32], resulting in 168 contigs (166 contigs within one scaffold, plus two additional short ones). This assembly covered 237816 aligned 454 reads (46535621 bases, 9.3-fold coverage) and 1616398 aligned Illumina reads (1616398 reads, 134499216 bases, 26.8-fold coverage). We aimed to close the gaps in the 454 assembly using PCR amplification and Sanger sequencing of gap regions. We therefore designed sequencing primers using primer3 [33]. For some gaps, the sequence was unobtainable. Sanger sequencing of PCR products was discontinued when the PacBio assembly became available. The 454 contigs were ordered and combined into a single contig by insertion of poly-N sequences of appropriate length.

### Comparison of genome assemblies

The PacBio and 454 assemblies were compared using an in-house script for analysis of near-identical genomes [9]. When an extended region of sequence identity was interrupted by poly-N stretches in the 454 assembly, we replaced this region by the corresponding sequence from the PacBio assembly. Similarly, discrepancies within repeat sequences (e.g. rRNA operons or transposons) were resolved. This improved 454 assembly allowed us to identify sequence differences in unique regions of the genome.

Illumina reads were mapped to both, the PacBio assembly and the improved 454 assembly (see below). All of the PacBio/454 discrepancies in unique regions were also detected by mapping of Illumina reads to the respective genome assemblies. Potential errors in the PacBio sequence were subjected to frameshift analysis via BLASTx comparison to UniProt.

### Error corrections using Illumina sequencing data

The correctness of the PacBio-derived sequence was validated by mapping of Illumina reads using three distinct strategies: (a) read mapping with TopHat [34] followed by mismatch and indel detection using SAMtools [35] and VarScan [36] (point mutations: pileup2SNP, indels: pileup2indel). For TopHat we used stringent mapping with the -g parameter set to “1”; for constructing the pileup file for VarScan we used SAMtools (mpileup) with the -A parameter to ensure that all reads were considered. This procedure was used to map Illumina reads to both, the PacBio and the 454 assemblies. (b) We also mapped Illumina reads using bowtie2 with default parameters [37]. This less stringent mapping procedure uncovered additional sequencing errors in one of the rRNA operons (operon J), while no additional differences were detected in the remainder of the genome. (c) As we identified additional sequencing errors by a less stringent mapping procedure, we became aware that both our Illumina read mapping strategies may be incomplete. Therefore, we developed a validation algorithm based on k-mer analysis (Fig. 1). We built independent catalogs of 49-mers from the genome and from the Illumina reads and compared those in two ways. We sought to identify frequently occurring read k-mers, which were not represented in the genome sequence. We also computed the read k-mer coverage at each genome position. Then, ***coverage drops*** and ***coverage slopes*** were computed using 24-mers (half of k-mer length-1). For coverage drops, the 24 upstream and downstream positions were considered (“inner” bases) as well as the adjacent 24 positions (“outer” bases). The coverage ratio of outer to inner bases defines the coverage drop. This is expected to be high if a divergent base is encountered. For coverage slopes, the “inner” bases are ignored and the coverage of the 48 adjacent bases upstream and downstream are considered. The ratio of left/right or right/left (whichever is above 1) is computed as coverage slope. This is expected to be high in case of a larger indel. This analysis uncovered further differences in rRNA operon J.

To investigate the co-occurrence of closely spaced polymorphic bases within repeat regions, we performed exhaustive string comparison using a simple text search in the Illumina readset. Sequences of 30–75 nucleotides (nt) were compared to the Illumina read set using string comparison (with the UNIX grep command). Correct combinations of polymorphic bases led to retrieval of many Illumina sequences, while mismatches of at least one base resulted in no or only a few retrieved sequences.

Following this procedure, we manually corrected the initial PacBio assembly, resulting in a final genome sequence of *A. salmonicida* subsp*. pectinolytica* strain 34mel (DSM 12609).

### Genome annotation

Automatic genome annotation was performed using the Rapid Annotation using Subsystem Technology (RAST) [11]. The Mummer ultra fast alignment algorithm [13] for large-scale DNA and protein sequences was used for the comparison to related strains.

The annotation was subjected to detailed manual curation. We extracted the proteomes of *A. salmonicida* subsp*. salmonicida* strain A449 and of *A. salmonicida* subsp*. pectinolytica* strain 34mel (derived from the draft genome) from UniProt. Also, the SwissProt section of UniProt (reviewed section, Dec-2015) was downloaded. We compared each protein to these three databases using BLASTp. (a) The start codon assignments were evaluated. For each BLASTp result with a closely related homolog, the alignment near the N-terminus was inspected, applying principles previously described [38]. When a problem case was encountered (e.g. when an ini-Met of one sequence aligned with an internal Met of the homolog), we attributed the problem to one of the aligned sequences. If attributed to our protein, the start codon assignment was corrected. To facilitate this analysis, we generated a custom script, which identified proteins, for which all closely related sequences had problem-free N-terminal alignments. These were considered valid and were excluded from manual curation. (b) For validation of protein names, genes and EC numbers, we applied the annotation strategy described for *Halomonas* [10]. We selected a close homolog from the pre-computed BLASTp result against SwissProt, preferably from *E. coli*. As SwissProt contains reviewed annotations, we considered it valid to copy this information. If a close homolog was lacking from SwissProt, we based our curation on other sources: more distant SwissProt homologs, homologs from *Halomonas elongata,* which we had extensively curated, InterPro domains, homologs from strains A449 and 34mel. If RAST had assigned a specific function while we did not identify a closely related and reliably annotated homolog with that function, we performed literature search with restricted effort. If this was also not successful, we assigned only a general protein name, consistent with our previous annotation attempts [10, 12].

RNA annotations can be based on various sources, which however suffer from inconsistent assignments of RNA termini. The RNA annotations are based on annotations for *A. salmonicida* subsp*. salmonicida* strain A449 (accession CP000644.1) by RFAM [39] and GtRNAdb [40]. These annotations were transferred to *A. salmonicida* subsp*. pectinolytica* strain 34mel genome by BLASTn analysis. The anticodon assignment of tRNA-SeC (ACA, decoding Cys) was taken from Mukai et al. [41]. The 16S rRNA 3′ end was taken from Shine and Dalgarno [42]. Many other sources have been inspected for RNA assignments (for more details, see Additional file 1: Text S1).

### Identification of transposons

A systematic search for transposons was performed by an iterative approach, based on the BLAST [43] suite of programs and the ISFinder database [19]. During this analysis, an exhaustive set of transposons and remnants thereof as found in various complete or draft *Aeromonas* genomes was accumulated and used as an in-house database for subsequent BLAST analyses. The in-house set of transposons was initialized by all transposons assigned by ISFinder to one of the *Aeromonas* species; and by all transposons that had been encountered upon validation of the genome assembly and upon comparison to the previously published draft genome of strain 34mel. Newly identified complete transposons (with both termini defined and with a non-interrupted transposase gene) were submitted to and accepted by ISFinder. If none of the finished genomes contained a complete transposon, the search was extended to draft genomes using BLASTn against the Whole Genome Sequencing (WGS) set at NCBI with a restriction to gamma-proteobacteria.

For further transposon analysis, a mapping file containing the complete genome sequence was prepared. In this file, transposons were annotated, splitting the genome into transposons and regions in between. These interjacent regions were subjected to further BLASTn analysis against the in-house transposon set until all transposons were assigned. To generate an even more exhaustive transposon set, interjacent regions were compared by both, BLASTn and BLASTx to all transposons stored in ISFinder. If not evident from BLAST analysis, transposon termini were identified by detection of inverted terminal repeats and of target duplications. In several cases, transposons were found to be interrupted and the intervening sequences were subsequently identified to represent additional targeting transposons.

In addition to our own strain, we systematically analyzed the mobilome of the following *Aeromonas* genomes (as identified by their GenBank accessions): *A. salmonicida* subsp*. salmonicida* strain A449 (chromosome and 5 plasmids, CP000644, CP000645, CP000646, AY301063, AY301064, AY301065), *A. media* strain WS (chromosome and 1 plasmid, CP007567, CP007568), *A. veronii* strain B565 (CP002607), *A. hydrophila* subsp. *hydrophila* ATCC 7966 (type strain, CP000462), *A. hydrophila* subsp. *hydrophila* strain AL06–06 (chromosome and 3 plasmids, CP010947, CP010948, CP010949, CP010950).

## Additional file

Additional file 1: **Figure S1.** Sequencing and assembly strategy of the *A. salmonicida* subsp. *pectinolytica* strain 34mel genome. **Figure S2.** Gene similarity and Mummer alignments to other *Aeromonas* genomes. **Figure S3.** Transposon conglomerates from *A. salmonicida* subsp. *pectinolytica* strain 34mel. **Figure S4.** Transposon family content in analyzed *Aeromonas* strains. **Figure S5.** Comparison of the *A. salmonicida* subsp. *pectinolytica* strain 34mel genome, plasmid pFBAOT6 from *Aeromonas caviae*, and transposon Tn1721. **Table S1.** Pathogenic as well as temperature-dependent growth features of analyzed *Aeromonas* strains. **Table S2.** General genome features of *A. salmonicida* subsp. *pectinolytica* strain 34mel. **Table S3.** Polymorphic sites in rRNAs from *A. salmonicida* subsp. *pectinolytica* strain 34mel. **Table S4.** Genes disrupted by transposons in *A. salmonicida* subsp. *pectinolytica* strain 34mel*.* **Table S5.** Transposon details from analyzed *Aeromonas* strains. **Table S6.** Genes retained in transposon TnAs1 from the environmental IncP-9 TOL plasmid pWW0 from *Pseudomonas putida*. **Text S1.** Annotation details for RNA genes in the *A. salmonicida* subsp. *pectinolytica* strain 34mel genome. **Text S2.** Short unassigned contigs from the *A. salmonicida* subsp. *pectinolytica* strain 34mel draft genome. **Text S3.** Transposons: nomenclature and completeness. **Text S4.** On transposon counting and the meaning of “ISAs11”. **Text S5.** Insertions on pFBAOT6 in transposon Tn1721 and details of the regions shared with the *A. salmonicida* subsp. *pectinolytica* strain 34mel genome. **Text S6.** Details of PacBio library preparation, sequencing, and assembly of the genome from *A. salmonicida* subsp. *pectinolytica* strain 34mel. (PDF 1171 kb)

## Abbreviations

Bp: Base-pair
Kb: Kilo-bases
Mb: Mega-bases
PCR: Polymerase chain reaction
SMRT: Single-molecule real-time sequencing
