# Supplementary material for: The complete and fully assembled genome sequence of Aeromonas salmonicida subsp. pectinolytica and its comparative analysis with other Aeromonas species: investigation of the mobilome in environmental and pathogenic strains
Source: BMC Genomics. 2018 Jan 5;19:20. doi: 10.1186/s12864-017-4301-6 (PMC5756416; doi:10.1186/s12864-017-4301-6)

Supplementary Material

Supplementary Text

Supplementary Text S1: Annotation details for RNA genes in the *A. salmonicida* subsp. *pectinolytica* strain 34mel genome.

tRNAs were initially predicted by RAST [1] and subsequently revised to be consistent with the tRNAs assigned to *A.salmonicida* subsp*. salmonicida* strain A449 in GtRNAdb [2]. This involved systematic addition of the genome-encoded CCA trinucleotide and post-prediction of an initial version of tRNA-SeC. The final sequence of tRNA-SeC and its anticodon ACA, which leads to decoding of the Cys codon TGT, were taken from Mukai et al [3]. tRNA assignments were consistent with RFAM [4] (except that RFAM also excludes the 3' CCA).

The termini of rRNAs were annotated according to the RFAM annotation of the genome of strain A449 (RFAM data retrieved for CP000644.1). For rRNA annotations, we initially had considered the annotation by RAST, data from RNAmmer [5] and from 5SRNAdb [6], as well as the publications of Shine and Dalgarno [7] and of Lin et al [8]. The 16S rRNA 5' end was annotated according to RFAM (the RNAmmer annotation is 7 bases shorter, the RAST version is 8 bases shorter). The 16S rRNA 3' end was annotated using BLASTn [9] against an *E. coli* sequence version, which is consistent with the experimentally determined 16S rRNA 3' end [7], terminating 3 bases beyond the highly conserved CCTCC sequence. This is consistent with RFAM, 2 bases shorter than proposed by Lin et al., 5 bases longer than RNAmmer and 34 bases longer than RAST. Both 23S rRNA ends were annotated according to RFAM (which is consistent with RNAmmer and with RAST at the 3' end, but 2 bases longer than RNAmmer and 7 bases shorter than RAST at the 5' end). Both 5S rRNA ends were annotated according to RFAM (which is 2 bases shorter than RAST and RNAmmer at the 5' end; at the 3' end, it is 1 base shorter than RAST and 3 bases longer than RNAmmer). The 33 *Aeromonas* 5S rRNA sequences in 5SrRNAdb show terminal heterogeneity.

The RNAseP RNA was annotated according to RFAM after an initial assignment using the bcheck server (http://rna.tbi.univie.ac.at/bcheck) [10]. The signal recognition particle RNA was annotated according to RFAM after an initial annotation using the SRP-Scan server (http://bio.lundberg.gu.se/srpscan) with parameters "Eubacteria common:

GRRA tetraloop, without Alu" [11]. The tmRNA was found to be nearly identical to that of *A. hydrophila* strain ATCC 7966, which was taken from the tmRDB database (http://rth.dk/resources/rnp/tmRDB) [12] and is consistent with RFAM. Both species encode the same tag peptide (ANDENYALAA**). The 6S RNA was annotated according to that from strain A449 [13] which is consistent with RFAM. Five sRNAs (rhyB, csrB, csrC, t44, spot42) were annotated according to RFAM. Initially, these were identified by BLASTn using the GenBank annotation of the A449 genome (accession CP000644). Except for spot42 RNA, the RFAM annotation showed considerable deviation in assignment of termini and was preferred over the GenBank annotation.

Supplementary Text S2: Short unassigned contigs from the *A. salmonicida* subsp. *pectinolytica* strain 34mel draft genome.

Contig ARYZ02000167 (1449 bp) is closely related (98% sequence identity) to several strains of *A. media*. Contig ARYZ02000209 (424 bp) is closely related (95% sequence identity) to a sequence from *A. piscicola* and to other subspecies of *A. salmonicida* (*A. salmonicida* subsp. *masoucida*, *A. salmonicida* subsp. *achromogenes*). Contig ARYZ02000165 (1591 bp) consists largely of transposon ISAeca3.

Supplementary Text S3: Transposons: nomenclature and completeness.

Transposons with the same name are very closely related (commonly at least 95% sequence identity) to follow ISFinder rules [14]. Otherwise, they are considered "distinct transposons". Transposon names are described in more detail below. Related transposons are assigned to the same “transposon type”. We mainly request at least a moderate protein sequence similarity of the transposase to assign transposons to the same type. There is no request that the DNA sequences are similar enough to allow cross-identification by BLASTn analysis.

Transposon nomenclature is based on ISFinder rules [14] for elements suitable for that database. For other transposons, an *ad hoc* nomenclature is used, which is similar to that used for halophilic archaea [15].

Transposons are suitable for ISFinder only if they are perfect. The element must be complete at both termini, must carry a transposase gene, and this gene must not show any evidence of gene disruption, such as in-frame stop codons, frameshifts, or premature termination.

ISFinder names are based on the species name followed by a serial number. Historical elements are an exception to this rule. Currently, a three-letter abbreviation is assigned to each species (e.g. ISAve for ***A***_eromonas_ ***ve***_ronii_). If the corresponding three-letter combination is already in use, a four-letter abbreviation is assigned (e.g. ISAeme for ***Ae***_romonas_ ***me***_dia_). Historic transposons may have a name, which is not based on a species name (e.g. IS5) or where only two letters are used to indicate the species (e.g. ISAs for ***A***_eromonas_ ***s***_almonicida_). Once a transposon name is assigned, this name is maintained even if the basis for this assignment becomes invalid (e.g. ISApu1 is from *Aeromonas caviae*, formerly known as ***A***_eromonas_ ***pu***_nctata_). More recent elements are based on the revised species name (e.g. ISAeca1 from ***Ae***_romonas_ ***ca***_viae_).

In ISFinder, all transposons, which have less than 5% sequence difference are considered the same transposon, even if they are found in distinct species. Thus, *A. salmonicida* contain copies of the transposons IS5 and ISAhy1. To clarify this issue, we refer to these as Aersa_IS5 and Aersa_ISAhy1, the prefix Aersa_ being used for *A. salmonicida* subsp. *pectinolytica* strain 34mel. We use e.g. As449_ as prefix for *A. salmonicida* subsp*. salmonicida* strain A449.

Different copies of the same transposon are indicated by one serial letter (from _A onwards) or two serial letters (from _AA onwards, if more than 26 copies are found in a genome). If an element is split into several fragments, which is commonly caused by suffering an insertion of another transposon, the fragments receive serial numbers, with 1 being the most 5' fragment.

Our definition of a "complete" element differs from that of ISFinder as we only consider the element termini. Thus, an element with complete termini but a large internal deletion is considered complete in this manuscript, while it would be considered disrupted by ISFinder. We use ISFinder style names for incomplete elements, if the sequence similarity to a complete element is high enough.

MITEs (Miniature Inverted-Terminal repeat Elements) are mobile elements, which are devoid of a transposase gene. A section for MITEs was only recently added to the ISFinder database. Elements are named according to the MITE naming rules of ISFinder.

Elements, which are incomplete at one or both termini, or elements, which have a disrupted transposase gene are not themselves suitable for ISFinder. We have made an attempt to identify a suitable element by BLASTn analysis of the NCBI nr database or the gamma-proteobacteria subsection of NCBI's WGS (whole-genome shotgun) database. If these attempts were not successful, an element suitable for ISFinder cannot be identified. These elements were also named according to an *ad hoc* nomenclature [15].

Ad hoc names are built from a strain-specific abbreviation, followed by the term IRS (IS-related sequence) and a serial number. We use AsIRS for elements from *A. salmonicida* subsp. *pectinolytica* strain 34mel, As449IRS for elements from *A. salmonicida* subsp. *salmonicida* strain A449, etc.

Supplementary Text S4: on transposon counting and the meaning of "ISAs11"

We analyzed and counted mobile genetic elements in various strains of *Aeromonas*. Likewise, Vincent *et al.* also reported transposon frequency analyses [16]. For various reasons detailed below, the results of the two studies are not well comparable.

First, two different methods for transposon counting were used. We analyzed strains with a complete, final genome sequence and count every occurrence of a transposon as one element. In contrast, Vincent *et al.* count mapped sequence reads to a selection of IS elements. In this case, results can vary considerably for strains, which contain high copy-number plasmids: we count the elements once, while the counts in Vincent *et al.* are proportional to the plasmid's copy number.

The methodological differences also preclude that the same set of strains can be analyzed. Our analysis is restricted to strains with a complete, finalized genome sequence. Most of the strains analyzed by Vincent *et al.* are thus not suitable for our analysis. The approach, which Vincent *et al.* have taken, requires access to raw sequence read data. Such reads have typically not been deposited for completely sequenced genomes. Finally, it should be noted that *A. salmonicida* subsp. *salmonicida* as analyzed by Vincent *et al.* refers to strain RS526, while we refer to the strain A449.

Because of this difference in the methodological approach, data from this project and the report from Vincent and colleagues are not truly comparable.

We note that the two studies show one clear discrepancy: according to Figure 4 in Vincent *et al.*, transposon ISAs11 is frequent in psychrophilic strains, including *A. salmonicida* subsp. *salmonicida,* but is lacking in *A. salmonicida* subsp. *pectinolytica*. In our analysis, ISAs11 has not been identified at all, neither in *A. salmonicida* subsp. *salmonicida* nor in *A. salmonicida* subsp. *pectinolytica*. We attempted to reconcile this discrepancy and identified severe annotation problems with respect to "ISAs11". There is confusion between ISAs11 and ISAs3, which seems to reoccur in *Aeromonas* annotations and publications:

ISAs11 is ISAs3 according to ISFinder. ISFinder (accessed 21-Sep-2017) lists ISAs11 as one of the transposons from *Aeromonas salmonicida* as submitted by Michael Reith. The underlying link opens the details page for ISAs3, a 1326 bp element, which belongs to family IS256.

ISAs11 is not ISAs3 according to the publication of Reith *et al.*. The genome of *A. salmonicida* subsp. *salmonicida* strain A449 was sequenced by Reith *et al.* [17]. In Table 2 of that publication, ISAs3 is listed as a 1326 bp element, which belongs to the IS256 family. In contrast, ISAs11 is listed as a 2614 bp element, which belongs to family IS21. Clearly, ISAs3 and ISAs11 as described in Reith *et al.* are distinct and unrelated transposons.

ISAs11 is ISAs3 according to the genome submitted by Reith *et al.*. In the GenBank entry of the strain A449 genome, submitted by Reith *et al*. (accession CP000644, accessed 21-SEP-2017), there is only a single annotation named "ISAs11" and this is a copy of the 1326 bp element ISAs3. Similarly, the annotated ISAs11 in strain A449 plasmid 5 (accession CP000646) is a copy of ISAs3.

ISAs11 is used as an equivalent of ISAs3 in subsequent publications. A rearrangement was detected in a copy of this plasmid (pAsa5 in strain 01-B526, accession KY555069) and was described to be caused by "IS11 from the IS256 family" [18], again referring to the transposon listed as ISAs3 and not to the transposon listed as ISAs11 in Reith *et al.* Also, in Vincent *et al.*, ISAs11 is made responsible for one key gene inactivation event, which is assigned to the ancestor of the psychrophilic strains. When

describing ISAs11, the authors refer to plasmid pAsal1 from strain 01-B526 (Genbank Accession: AJ508382, [19]). This plasmid contains a single transposon, namely ISAs3.

Transposon labeling ambiguities in Vincent *et al.*. Unfortunately, Figure 4 of Vincent *et al.* shows not only ISAs11 (which refers to ISFinder ISAs3) but also another transposon labeled ISAS3. According to the annotation in CP000646, the transposon type ISAS3 refers to ISFinder ISAs7. Likewise, two of the transposons are labeled ISAs4 and ISAS4. ISAs4 refers to ISFinder ISAs4. In CP000644, the transposon type ISAS4 refers to the ISFinder transposon ISAhy2 or targeted versions thereof. The transposon type ISAS5 annotations are heterogeneous in length but most cover ISAs29 and thus the originally attributed ISAs11 (see below), but frequently continue into and cover adjacent, unrelated transposons. With respect to ISAs3, we find 5 complete copies in *A. salmomicida* subsp. *salmonicida* strain A449 but only a one in *A. salmomicida* subsp. *pectinolytica* strain 34mel. If this transposon is the one labeled as ISAs11 in Figure 4 in Vincent *et al.*, our data are consistent.

ISAs29 is most likely the transposon originally referred to as ISAs11. The transposon named ISAs11 in Reith *et al.*, Table 2, belongs to the IS21 family, is 2614 bp long, and is reported to have 12 complete plus 3 partial copies in the strain A449 genome. These data are near-identical to the transposon, which we have submitted to ISFinder as ISAs29. This element belongs to the IS21 family, is 2613 bp long, and we found 12 complete and 2 partial copies in the strain A449 genome. The 34mel genome also contains 4 complete copies of this transposon.

Supplementary Text S5: Insertions on pFBAOT6 in transposon Tn1721 and details of the regions shared with the *A. salmonicida* subsp. *pectinolytica* strain 34mel genome.

Transposon Tn1721 [20] consists of a "basic transposon" (Tn1722, 5.4 kb) with strong inverted terminal repeats, and an extension of 5.3 kb with a 3rd copy of the inverted terminal repeat. Plasmid pFBAOT6 from *A. caviae* has a complete copy of transposon Tn1721 with two insertions of 3415 bp and of 28247 bp.

The 3.4 kb insertion consists of a transposon cassette with oppositely oriented ISApu2 and ISApu1 transposons, which have related terminal repeats, separated by a unique

sequence of 642 bp. This cassette is bounded by a 10 bp direct repeat, indicating that it has been mobilized in one step. It targets the transposase gene from Tn1722 on pFBAOT6. The genome of *A. salmonicida* subsp*. pectinolytica* strain 34mel contains an exact copy of this insert and the adjacent transposase gene as part of a 22,017 bp segment of complete sequence identity.

The 28 kb insert in pFBAOT6 Tn1721 starts right after the "basic transposon" Tn1722, separating it from the Tn1721-specific extension. This insert terminates with a sequence that has only 3 point mutations to the 6.5 kb transposon ISPa38, which has similarity to Tn3-type transposons.

A. salmonicida subsp*. pectinolytica* strain 34mel shares 26606 bp of complete sequence identity in 4 sections with pFBAOT6, the longest section having 22017 bp. It starts within the "basic transposon" Tn1722, includes the complete 3.4 kb Apu2/Apu1 cassette and continues for 14 kb into the 28 kb insert. At this point is a pFBAOT6-specific insertion (a 206 bp MITE, submitted to ISFinder as MITEAeca1 and closely related to MITEAeme1), followed by the 2nd shared section of only 611 bp. At this point are unrelated sequences of 1865 bp in pFBAOT6 and of 1031 bp, coding for a resolvase, in strain 34mel. The 3rd shared segment is also short with only 590 bp. On pFBAOT6, the remainder of the 28 kb insert consists of a 3585 bp region followed by a complete copy of the 6.4 kb transposon ISPa38. Of this, 3400 bp are also found in the strain 34mel genome as the 4th shared section (named AsIRS13). The Tn1721-specific extension, which follows on plasmid pFBAOT6, is not present in the strain 34mel genome. This extension codes for tetracyclin resistance genes.

Only part of the "basic transposon" Tn1722 is shared between pFBAOT6 and the 34mel genome. The Tn1722 -specific sequence codes for a gene, which is related to methyl-accepting chemotactic proteins and has been shown to interfere with chemotaxis upon overexpression [20]. In the strain 34mel genome, this is replaced by a different set of passenger genes so that TnAs1, a distinct complete transposon is formed. The TnAs1-specific sequence is near-identical to a region from the IncP-9 TOL plasmid pWW0 from *Pseudomonas putida* (Supplementary Table S6). This exemplifies the high plasticity of Tn3-type transposons.

Supplementary Text S6: Details of PacBio library preparation, sequencing, and assembly of the genome from *A. salmonicida* subsp. *pectinolytica* strain 34mel.

library preparation and sequencing**:** 13 kb long insert libraries were prepared for PacBio sequencing according to the manufacturers protocols with slight modifications. Due to the size distribution of the gDNA after isolation, the DNA was directly subjected to the Library Preparation. The final long insert PacBio libraries were size selected for fragments larger than 7 kb using the BluePippin device. PacBio SMRT sequencing was performed with the P4/C2 chemistry and 180 minutes sequencing resulting in 118 K raw subreads, which were subjected to the assembly processing.

PacBio de novo assembly*:* After filtering for quality (>= 80 % accuracy, >500 bp length), the remaining 87215 reads (641.7 Mbp) with a mean read length of 7358 bp were used for the assembly with the PacBio SMRTanalysis pipeline. The assembly was done using HGAP (DAGCON-based hierarchical genome assembly process, version RS_HGAP_assembly.2) following the steps pre-assembly, *de novo* assembly with the Celera assembler and final polishing with Quiver (consenus and variant calling). We obtained two contigs, one of 5036397 bp size and a 110-fold mean coverage and a second poorly covered contig of 6568 bp in size. The 110-fold mean coverage is somewhat lower than the purely computational coverage of 127-fold (641.7 Mb for a 5.036 Mb contig).

Supplementary Tables

Species	(P)athogenic/ (N)on-pathogenic	(M)esophilic/ (P)sychrophilic
A. s. salmonicida	P	P
A. s. pectinolytica	N	M
A. media	N	M
A. hydrophila 7966	P	M
A. hydrophila AL06	P	M

Supplementary Table S1: Pathogenic as well as temperature-dependent growth features of analyzed *Aeromonas* strains.

Genomic feature	final	initial (RAST)
replicons	1	
chromosome length	5012649	
G+C content	58.3%	
Protein coding genes	4590	4502
Genes in RAST subsystems		2334
Disrupted genes	209	
RNA genes (total)	165	155
rRNA operons	10	
tRNA_genes	125	124
Other_RNA_genes	9	

Supplementary Table S2: General genome features of *A. salmonicida* subsp. *pectinolytica* strain 34mel. For protein-coding and RNA genes, data are provided for the final annotation and the initial RAST annotation.

		operon											
16S rRNA	pos	A	B	C	D	E	F	G	H	I	J		
		1	2	2	3	1	4	5	6	7	4		version
	212	C	C	C	C	C	C	C	A	C	C		
	461	GCG	GCG	GCG	GCG	GCG	GCG	GCG	GCG	ATA	GCG		
	1006	A	A	A	G	A	A	A	A	G	A		
	1011	TGC	CCA	CCA	TGT	TGC	TGT	CCA	TGC	TGC	TGT		
	1020	GC	TG	TG	AC	GC	AC	TG	GC	AC	AC		
	1138	T	C	C	T	T	T	T	T	T	T		
		operon											
23S rRNA	pos	A	B	C	D	E	F	G	H	I	J		
		1	2	3	4	2	5	6	2	7	8		version
	137	GC	GC	GC	GC	GC	GC	AG	GC	GC	GC		
	140	C	C	C	C	C	C	T	C	C	C		
	143	GC	GC	GC	GC	GC	GC	CT	GC	GC	GC		
	152	CTAT	CTAT	CTAT	CTAT	CTAT	CTAT	CGT	CTAT	CTAT	CTAT		
	156	A	A	A	G	A	A	A	A	A	A		
	158	CT	CT	CT	CT	CT	CT	CAT	CT	CT	CT		
	169	GG	GG	GG	GG	GG	GG	GTG	GG	GG	GG		
	172	T	T	T	C	T	T	T	T	T	T		
	174	ATAG	ATAG	ATAG	ATAG	ATAG	ATAG	ACG	ATAG	ATAG	ATAG		
	1158	T	T	C	T	T	T	T	T	C	C		
	1167	A	A	G	A	A	A	A	A	G	G		
	2826	C	T	T	T	T	T	T	T	T	C		
	2839	T	C	C	C	C	C	C	C	C	T		
	2842	A	A	G	A	A	G	A	A	A	A		
	2844	C	T	C	T	T	C	T	T	T	C		
	2847	T	T	C	T	T	C	T	T	T	T		
	2849	CA	TG	TG	TG	TG	TG	TG	TG	TG	CA		
	2863	G	A	A	A	A	A	A	A	A	G		
		operon											
5S rRNA	pos	A	B1	C	D	E	F	G	H	I	J	B2	
		1	1	1	1	1	1	1	1	1	1	2	version
	5	G	G	G	G	G	G	G	G	G	G	A	
	10	C	C	C	C	C	C	C	C	C	C	A	
	18	C	C	C	C	C	C	C	C	C	C	T	
	61	G	G	G	G	G	G	G	G	G	G	A	
	110	C	C	C	C	C	C	C	C	C	C	T	

Supplementary Table S3: Polymorphic sites in rRNAs from *A. salmonicida* subsp. *pectinolytica* strain 34mel. For each of the rRNAs (16S, 23S, 5S) each of the 10 operons (A to J) is analyzed and all polymorphic positions are listed (pos). Underneath the operon letters is a version number to highlight sequence identity between operons. If polynucleotides are reported, the position refers to the 1st base. As is typical for many

organisms, each operon has a "typical" version of the 5S rRNA, directly following the 23S rRNA. One of the operons contains a tandem pair of 5S rRNAs, the 2nd being "atypical" and divergent from all the others. The typical 5S rRNA of operon B is referred to as B1, the atypical as B2.

code	category	transposon	protein name
Asalp_02710	targeted	ISAs31	RND family transport protein MFP component
Asalp_08430		ISAs20	argininosuccinate lyase
Asalp_10850		ISAs1	response regulator / EAL-type diguanylate phosphodiesterase
Asalp_11150		ISAs23	HD-GYP family diguanylate phosphodiesterase
Asalp_11920		ISAs30	EAL-type diguanylate phosphodiesterase
Asalp_11960		IS5	conserved hypothetical protein
Asalp_12690		ISAs25	conserved hypothetical protein
Asalp_14140		ISAs13	conserved hypothetical protein
Asalp_14720		ISAs12	transposase domain protein
Asalp_17870		ISAs27	exotoxin A
Asalp_19640		ISAs21	conserved hypothetical protein
Asalp_19870		IS5	DUF2931 family protein
Asalp_20100		ISAs27	HD family hydrolase domain protein
Asalp_20130		ISAs26	conserved hypothetical protein
Asalp_20360		ISAs12	amidohydrolase domain protein
Asalp_21450		ISAs28	LuxR family transcription regulator
Asalp_21620		ISAs20	O-antigen ligase domain protein
Asalp_22030		IS5	alanine dehydrogenase domain protein
Asalp_22750		ISAs2	conserved hypothetical protein
Asalp_22860		IS5	probable potassium-efflux system protein
Asalp_23850		ISUnCu16	peptidase M17 family protein
Asalp_24020		ISAs2	thiol:disulfide interchange protein DsbA
Asalp_25320		ISKpn10	DUF3330 domain protein / EAL-type diguanylate phosphodiesterase
Asalp_25400		TnAs2	SRAP domain protein
Asalp_26370		ISAs1+ISA	s27 glycoside hydrolase domain protein
Asalp_27540		ISAs20	integrase family protein
Asalp_27850		IS5	conserved hypothetical protein
Asalp_28190		ISAs2	LURP1 domain protein
Asalp_28290		ISAs1	cytochrome domain protein
Asalp_28770		ISAs12	PHP domain protein
Asalp_29110		ISAs12	hydrogenase 2 small subunit
Asalp_29840		ISUnCu16	TPR domain protein
Asalp_30400		ISKpn10	DUF3302 family protein
Asalp_31820		ISAs29	Hcy-binding domain protein
Asalp_32360		ISAs30	conserved hypothetical protein
Asalp_32490		ISAs12	wzz domain protein
Asalp_32880		ISKpn3	conserved hypothetical protein
Asalp_35660		ISAs14	PilM-type fimbrial assembly protein
Asalp_39660		ISAhy2+IS	As18 conserved hypothetical protein
Asalp_39740		ISAs12	ComFB domain protein
Asalp_40390		ISAhy2	pilus biogenesis protein TapB
Asalp_41240		ISAhy2+IS	Apu2+ISAs20 integrase family protein
Asalp_41440		ISAs20	diguanylate cyclase
Asalp_42790		ISAs18	methyl-accepting chemotaxis sensory transducer
Asalp_43750		IS5	conserved hypothetical protein

Asalp_45180		IS5	condensation domain protein AsbB
Asalp_20620	targeted and inverted	ISAs27	probable dioxygenase
Asalp_21800		ISAs27	probable dioxygenase
Asalp_20650		ISAs19	DUF4365 domain protein
Asalp_30800		ISAs19	DUF4365 domain protein
Asalp_39270		ISAs27	diguanylate cyclase
Asalp_39690		ISAs27	diguanylate cyclase
Asalp_07330	truncated	IS5	conserved hypothetical protein
Asalp_07390		IS5	HAE1 family transport protein (probable substrate copper)
Asalp_07410		IS5	copper resistance protein PcoB
Asalp_07450		ISAs27	probable molybdopterin-dependent oxidoreductase
Asalp_08410		IS5	deoxyguanosine triphosphate triphosphohydrolase
Asalp_08920		ISAs1	conserved hypothetical protein
Asalp_09140		IS5	DNA-binding protein H-NS
Asalp_09210		AsIRS20	conserved hypothetical protein
Asalp_12660		IS5	ABC-type transport system ATP-binding protein (probable substrate copper)
Asalp_12680		IS5	conserved hypothetical protein
Asalp_19890		ISAs7	DUF2235 domain protein
Asalp_20170		AsIRS9	DUF4258 family protein
Asalp_20210		IS5	probable ATP-dependent helicase
Asalp_20420		ISAs29	DnaJ domain protein
Asalp_20860		ISAs17	YhcC family protein YhcC
Asalp_21560		ISAs1	DUF2235 domain protein
Asalp_21630		ISAs21	wzz domain protein
Asalp_21720		IS5	TPR domain protein
Asalp_26310		ISKpn10	diguanylate cyclase
Asalp_26740		IS5	phage terminase domain protein
Asalp_26760		IS5	hypothetical protein
Asalp_27370		ISAs20	delta-endotoxin
Asalp_27390		ISAs20	conserved hypothetical protein
Asalp_27830		AsIRS3	conserved hypothetical protein
Asalp_31330		ISAs20	NERD domain protein
Asalp_37670		IS5	thiolase domain protein
Asalp_41590		IS5	nucleotidase domain protein
Asalp_45740		IS5	hypothetical protein

Supplementary Table S4: Genes disrupted by transposons in *A. salmonicida* subsp. *pectinolytica* strain 34mel. List of pseudogenes, which have been generated by transposon targeting. Code is the assigned ordered locus tag. If a protein-coding gene is targeted by a transposon, it is split into two or more fragments. For genes in category "targeted", there is no further rearrangement. We assign a single locus tag, thus annotating a single multi-region ORF. Category "targeted and inverted" is assigned, if the

targeting transposon is involved in a genome inversion. In this case, the gene fragments become disconnected (see Figure 4). Each gene fragment is assigned its locus tag. Category "truncated" is assigned when the targeting transposon is subsequently involved in another genome rearrangement. As a result, part of the gene is lost.

type	name	*A. s. pectinylotica*	*A.s. salmonicida*	*A. media*	*A. veronii*	*A. hydrophila 7966*	*A. hydrophila AL06*	family	status
IS5	IS5	37/30	-	30/28	-	-	-	IS5	+
IS26	IS26	-	-	1/0	-	-	2/2	IS6	+
ISAhy1	ISAhy1	-	-	-	-	2/2	8/8	IS1595	+
ISAhy1	ISAs23	1/1	-	1/0	-	-	-	IS1595	s
ISAhy1	ISAeme10	-	-	1/1	-	-	-	IS1595	s
IS630	ISAhy2	11/9	39/35	5/4	-	-	-	IS630	+
IS630	ISAeme16	-	-	2/1	-	-	-	IS630	s
IS30	ISAhy3	-	2/2	-	-	-	-	IS30	+
IS30	ISAs2	6/6	5/5	28/25	-	-	-	IS30	+
IS30	ISAeme18	-	-	1/1	-	-	-	IS30	s
IS30	ISAeca1	1/0	-	13/13	-	-	-	IS30	+
ISAs3	ISAs3	1/1	6/5	1/1	-	-	-	IS256	+
ISApu1	ISApu1	2/2	-	1/1	-	-	-	IS4	+
ISApu1	ISApu2	3/3	-	26/23	-	-	-	IS4	+
ISApu1	ISAeme3	-	-	3/2	-	-	-	IS4	s
ISApu1	ISAeme4	-	-	2/2	-	-	-	IS4	s
ISKpn3	ISKpn3	4/4	-	4/4	-	-	-	IS1595	+
ISKpn3	ISAeca5	-	-	4/4	-	-	-	IS1595	+
ISAs1	ISKpn9	-	-	10/9	-	-	-	ISAs1	+
ISAs1	ISAs1	11/7	2/2	13/8	-	-	-	ISAs1	+
ISAs1	ISAeme2	-	-	2/2	-	-	-	ISAs1	s
ISAs1	MITEAeme2	-	-	3/3	-	-	-	ISAs1	s
ISAs1	ISAs12	8/8	-	17/16	-	-	-	ISAs1	+
ISAs1	ISAeme1	-	-	7/5	-	-	-	ISAs1	s
ISKpn10	ISKpn10	6/4	-	2/1	-	-	-	IS3	+
ISKpn10	ISAs22	3/2	-	2/1	-	-	-	IS3	s
ISKpn10	ISAeme6	-	-	1/1	-	-	-	IS3	s
ISKpn15	ISUnCu16	5/5	-	5/4	-	-	-	IS66	+
ISKpn15	ISAs21	3/3	-	35/31	-	-	-	IS66	s
ISKpn15	ISAeme23	-	-	2/2	-	-	-	IS66	s
ISKpn15	ISAeme24	-	-	3/1	-	-	-	IS66	s
ISEc9	ISEc9	-	-	-	-	-	1/1	IS1380	+
IS903	ISEc35	-	-	9/9	-	-	-	IS5	+
IS903	ISAs13	5/4	-	-	-	-	-	IS5	s
IS903	ISAs14	1/1	-	-	-	-	-	IS5	s
IS903	ISAs15	1/1	-	-	-	-	-	IS5	s
IS903	ISAeme7	-	-	1/1	-	-	-	IS5	s
IS903	ISAve2	-	-	-	1/1	-	-	IS5	s
ISPa40	TnAs1	1/1	-	-	-	-	-	Tn3	s
ISPa40	TnAs2	1/1	-	-	-	-	-	Tn3	s
ISPa40	TnAs3	-	1/1	-	-	-	-	Tn3	s
IS3	ISAs6	-	6/6	-	-	-	-	IS3	+
IS3	ISAs33	-	1/1	-	-	-	-	IS3	s

IS3	ISAve4	-	-	-	4/3	-	-	IS3	s
IS3	ISAs7	1/0	14/13	-	-	-	-	IS3	+
IS3	ISAs32	-	1/1	-	-	-	-	IS3	s
IS3	ISAeme20	-	-	2/2	-	-	2/2	IS3	s
ISAs8	ISAs8	-	1/1	-	-	-	-	IS1	+
IS1240	ISAs9	-	4/4	-	-	-	-	IS3	+
IS1240	ISAs20	8/8	-	4/4	-	-	-	IS3	s
IS1328	ISAs16	1/1	-	-	-	-	-	IS110	s
IS1328	ISAve1	-	-	-	3/3	-	-	IS110	s
IS1328	ISAs24	10/10	-	-	-	-	-	IS110	s
IS2	ISAs17	3/3	-	-	-	-	-	IS3	s
IS2	ISAve3	-	-	-	2/1	-	-	IS3	s
ISAs18	ISAs18	9/8	-	12/10	-	-	-	IS4	s
IS1419	ISAs19	6/4	5/0	-	-	-	-	IS481	s
ISShes12	ISAs25	2/2	-	7/5	-	-	-	IS1634	s
IS1341	ISAeme9	-	-	4/4	-	-	-	IS200/IS605	s
IS605	ISAs26	1/1	-	9/9	-	-	-	IS200/IS605	s
IS605	ISAeme8	-	-	7/5	-	-	-	IS200/IS605	s
ISEc12	ISAs27	14/14	-	-	-	-	-	IS21	s
ISEc12	ISAs28	2/2	-	8/7	-	-	-	IS21	s
ISEc12	ISAs29	5/4	14/12	3/3	-	-	-	IS21	s
IS4	ISAs30	16/16	-	-	-	-	-	IS4	s
IS4	ISAeme12	-	-	2/2	-	-	-	IS4	s
IS4	ISAeme13	-	-	1/1	-	-	-	IS4	s
IS4	ISAeme14	-	-	1/1	-	-	-	IS4	s
IS4	ISAeme15	-	-	36/35	-	-	-	IS4	s
IS4	MITEAeme1	-	-	3/3	-	-	-	IS4	s
IS4	MITEAve1	-	-	-	1/1	-	-	IS4	s
ISSen1	ISAs31	3/3	-	-	7/6	-	-	IS3	s
ISAs34	ISAs34	-	1/1	-	-	-	-	IS5	s
ISAeme5	ISAeme5	-	-	1/1	-	-	-	IS66	s
ISAeme11	ISAeme11	-	-	1/1	-	-	-	IS701	s
ISPsy4	ISAeme17	-	-	1/1	-	-	-	IS21	s
IS1001	ISAeme19	-	-	2/2	-	-	-	ISL3	s
IS481	ISAeme21	-	-	4/4	-	-	-	IS481	s
IS50	ISAeme22	-	-	1/1	-	-	-	IS4	s

Supplementary Table S5: Transposon details from analyzed *Aeromonas* strains. The number of copies is provided for each distinct transposon as indicated by their name. Transposons belong to the same type when their transposases show considerable sequence similarity. For MITEs, this decision is based on the sequence of the inverted terminal repeats. At ISFinder, transposons are further grouped into families if there is at least marginal sequence similarity. Transposon types from the same family and

transposons from the same type are grouped in this table. For each of the analyzed genomes, the number of all copies and of complete copies is provided (with a slash separating the counts for all and complete, respectively). The absence of the transposon in a genome is indicated by a dash (-). Only transposons with ISFinder names are listed. For a definition of ISFinder names and of classification as complete see Supplementary Text S3. Many of the transposons listed here have been submitted to and accepted by ISFinder during this analysis (labeled “s” in the status column). Elements labeled “+” were in ISFinder prior to this project or were submitted by other groups thereafter.

code	domain name	PFAM number	description
Asalp_45687	osmC	PF02566	Osmotically inducible protein C (OsmC) (P23929) is a stress -induced protein found in *E. coli*. This family also contains a organic hydroperoxide detoxification protein (O68390) that has a novel pattern of oxidative stress regulation [21].
Asalp_45690	adh_short_C2	PF13561	The short-chain dehydrogenases/reductases family (SDR) [22] is a very large family of enzymes, most of which are known to be NAD- or NADP-dependent oxidoreductases.
Asalp_45693	TetR_N	PF00440	This entry represents a DNA-binding domain with a helix-turn-helix (HTH) structure that is found in several bacterial and archaeal transcriptional regulators, such as TetR, the tetracycline resistance repressor.
Asalp_45696	CUPIN_7 (2x)	PF12973	This clan represents the conserved barrel domain of the 'cupin' superfamily ('cupa' is the Latin term for a small barrel). The cupin fold is found in a wide variety of enzymes, but notably also contains the non-enzymatic seed storage proteins [23,24]. The cupin domain is also found in transcriptional activator ChrR [25] and other proteins.

Supplementary Table S6: Genes retained from the environmental IncP-9 TOL plasmid pWW0 from *Pseudomonas putida.*

Supplementary Figure Legends

Supplementary Figure S1: Sequencing and assembly strategy of the *A. salmonicida* subsp. *pectinolytica* strain 34mel genome. This figure illustrates that PacBio long read sequencing (left) resulted in a single contig. 454 sequencing resulted in 168 contigs which were ordered and some gaps were closed by generation and sequencing of PCR products (not shown). Prior to Illumina-based validation, the 454 assembly was enhanced (not shown; gaps were filled, initially by poly-N regions of the expected length, later with the corresponding sequence from the PacBio assembly). Illumina reads were mapped independently to the PacBio and to the enhanced 454 assembly. Using stringent read mapping parameters, Illumina-based validation detected 60 discrepancies to the 454 assembly and 8 discrepancies in the PacBio assembly.

Supplementary Figure S2: Gene similarity and Mummer alignments to other *Aeromonas* genomes. Panel (**A**) is taken from RAST analysis. It indicates the ORFs (protein-coding genes) in the strain 34 mel genome as compared to closely related *Aeromonas* genomes. ORFs are drawn as ticks according to their relative genome position in the 34mel genome. Each of the four related genomes is indicated by one circle (from outside to inside: *A. media* strain WS, *A. hydrophila* subsp. *hydrophila* ATCC 7966, *A. salmonicida* subsp. *salmonicida* strain A449, *A. veronii* strain B565). For each ORF having a homolog in the corresponding genome, a colored tick is drawn with the color indicating sequence identity. The strain A449 genome contains an especially high fraction of highly related genes. Panels (**B** to **D**): Mummer-based genome alignments. Red dots/lines indicate matches in forward orientation, blue dots/lines matches in reverse orientation.

Supplementary Figure S3: Transposon conglomerates from *A. salmonicida* subsp. *pectinolytica* strain 34mel. In each panel, a transposon conglomerate is schematically shown. Arrows indicate transposons and their orientation. The transposon type is indicated. Partial transposons are indicated by dashed lines. Internal ends of targeted transposon are indicated by small circles. The overall length of each of the transposon conglomerates is indicated.

Supplementary Figure S4: Transposon family content in analyzed *Aeromonas* strains. (**A**) Only complete transposons and (**B**) all transposons are shown. The sum of all transposon families in Supplementary Table S5 was built to visualize the difference in family content for the analyzed *Aeromonas* genomes.

Supplementary Figure S5: Comparison of the *A. salmonicida* subsp. *pectinolytica* strain 34mel genome, plasmid pFBAOT6 from *Aeromonas caviae* and transposon Tn1721. (**A**) This panel sketches regions, which are common with 100% sequence identity (blue, regions A-D), specific for plasmid pFBAOT6 (light-green, regions E-I) or specific for the 34mel genome (light-red, regions J-L). To fit into one line, the scaling is compressed 2-fold compared to panels **B**-**E**. (**B**) 44 kb from the 34mel genome are shown, with the genome indicated by the thick black line (drawn twice). Each of the adjacent open boxes represents a 1 kb region. Regions are indicated by colored boxes and correspond to those in panel A. Transposons are indicated by thin arrows. ORFs (protein-coding genes) are drawn by thick open or colored arrows. Striped ORFs specify pseudogenes; dark brown coloring indicates resolvase genes; ORFs colored according to their associated transposon are transposase genes; yellow, striped ORFs are transposase pseudogenes; ORFs colored red are highly homologous to proteins from the IncP-9 TOL plasmid pWW0 from *Pseudomonas putida* (Supplementary Table S6). The corresponding part of region L is also colored red (this region is not highlighted in panel A). Orange arrowheads adjacent to Apu1/Apu2 indicate a target duplication; this pair of transposons has probably been mobilized as a cassette; a co-mobilized intervening 642 bp region is indicated by the curved line. Yellow arrowheads in TnAs1 and AsIRS12 indicate three copies of a 38 bp inverted terminal repeat. Some functionally relevant ORFs are marked by gene symbols. (**C** and **D**) these panels show part of plasmid pFBAOT6. See panel **B** for an explanation of symbols. As region **D** is identical between plasmid pFBAOT6 and the 34mel genome, it is not redrawn to completion; instead, the central region is missing (indicated by vertical white lines). Plasmid pFBAOT6 carries a complete copy of transposon Tn1721 with two insertions. (**E**) This panel shows transposon Tn1721, which consists of a basic transposon (Tn1722) bounded by inverted terminal repeats (indicated by yellow arrowheads) and an extension, which carries tetracyclin resistance genes, a pseudogene with close similarity to the Tn1722 transposase, and a third copy of the inverted terminal repeat.

Supplementary References

1. Aziz RK, Bartels D, Best AA, DeJongh M, Disz T, Edwards RA, et al. The RAST Server: rapid annotations using subsystems technology. BMC Genomics. 2008;9:75.

2. Chan PP, Lowe TM. GtRNAdb 2.0: an expanded database of transfer RNA genes identified in complete and draft genomes. Nucleic Acids Res. 2016;44:D184–9.

3. Mukai T, Englert M, Tripp HJ, Miller C, Ivanova NN, Rubin EM, et al. Facile Recoding of Selenocysteine in Nature. Angew. Chem. Int. Ed. Engl. 2016;55:5337–41.

4. Nawrocki EP, Burge SW, Bateman A, Daub J, Eberhardt RY, Eddy SR, et al. Rfam 12.0: updates to the RNA families database. Nucleic Acids Res. 2015;43:D130–7.

5. Lagesen K, Hallin P, Rødland EA, Staerfeldt H-H, Rognes T, Ussery DW. RNAmmer: consistent and rapid annotation of ribosomal RNA genes. Nucleic Acids Res. 2007;35:3100–8.

6. Szymanski M, Zielezinski A, Barciszewski J, Erdmann VA, Karlowski WM. 5SRNAdb: an information resource for 5S ribosomal RNAs. Nucleic Acids Res. 2016;44:D180–3.

7. Shine J, Dalgarno L. The 3'-terminal sequence of Escherichia coli 16S ribosomal RNA: complementarity to nonsense triplets and ribosome binding sites. Proc Natl Acad Sci USA. 1974;71:1342–6.

8. Lin Y-H, Chang BCH, Chiang P-W, Tang S-L. Questionable 16S ribosomal RNA gene annotations are frequent in completed microbial genomes. Gene. 2008;416:44–7.

9. Altschul SF, Madden TL, Schäffer AA, Zhang J, Zhang Z, Miller W, et al. Gapped BLAST and PSI-BLAST: a new generation of protein database search programs. Nucleic Acids Res. 1997;25:3389–402.

10. Yusuf D, Marz M, Stadler PF, Hofacker IL. Bcheck: a wrapper tool for detecting RNase P RNA genes. BMC Genomics. 2010;11:432.

11. Rosenblad MA, Gorodkin J, Knudsen B, Zwieb C, Samuelsson T. SRPDB: Signal Recognition Particle Database. Nucleic Acids Res. 2003;31:363–4.

12. Andersen ES, Rosenblad MA, Larsen N, Westergaard JC, Burks J, Wower IK, et al. The tmRDB and SRPDB resources. Nucleic Acids Res. 2006;34:D163–8.

13. Wehner S, Damm K, Hartmann RK, Marz M. Dissemination of 6S RNA among bacteria. RNA Biol. 2014;11:1467–78.

14. Siguier P, Gourbeyre E, Varani A, Ton-Hoang B, Chandler M. Everyman's Guide to Bacterial Insertion Sequences. Microbiol Spectr. 2015;3:MDNA3–0030–2014.

15. Dyall-Smith ML, Pfeiffer F, Klee K, Palm P, Gross K, Schuster SC, et al. Haloquadratum walsbyi: limited diversity in a global pond. Lopez-Garcia P, editor. PloS one. 2011;6:e20968.

16. Vincent AT, Trudel MV, Freschi L, Nagar V, Gagné-Thivierge C, Levesque RC, et al. Increasing genomic diversity and evidence of constrained lifestyle evolution due to insertion sequences in Aeromonas salmonicida. BMC Genomics. 2016;17:44.

17. Reith ME, Singh RK, Curtis B, Boyd JM, Bouevitch A, Kimball J, et al. The genome of Aeromonas salmonicida subsp. salmonicida A449: insights into the evolution of a fish pathogen. BMC Genomics. 2008;9:427.

18. Tanaka KH, Dallaire-Dufresne S, Daher RK, Frenette M, Charette SJ. An insertion sequence-dependent plasmid rearrangement in Aeromonas salmonicida causes the loss of the type three secretion system. Hensel M, editor. PloS one. 2012;7:e33725.

19. Fehr D, Casanova C, Liverman A, Blazkova H, Orth K, Dobbelaere D, et al. AopP, a type III effector

protein of Aeromonas salmonicida, inhibits the NF-kappaB signalling pathway. Microbiology (Reading, Engl.). 2006;152:2809–18.

20. Allmeier H, Cresnar B, Greck M, Schmitt R. Complete nucleotide sequence of Tn1721: gene organization and a novel gene product with features of a chemotaxis protein. Gene. 1992;111:11–20.

21. Mongkolsuk S, Praituan W, Loprasert S, Fuangthong M, Chamnongpol S. Identification and characterization of a new organic hydroperoxide resistance (ohr) gene with a novel pattern of oxidative stress regulation from Xanthomonas campestris pv. phaseoli. J. Bacteriol. 1998;180:2636–43.

22. Jörnvall H, Persson B, Krook M, Atrian S, Gonzàlez-Duarte R, Jeffery J, et al. Short-chain dehydrogenases/reductases (SDR). Biochemistry. 1995;34:6003–13.

23. Dunwell JM, Purvis A, Khuri S. Cupins: the most functionally diverse protein superfamily? Phytochemistry. 2004;65:7–17.

24. Dunwell JM, Culham A, Carter CE, Sosa-Aguirre CR, Goodenough PW. Evolution of functional diversity in the cupin superfamily. Trends Biochem Sci. 2001;26:740–6.

25. Campbell EA, Greenwell R, Anthony JR, Wang S, Lim L, Das K, et al. A conserved structural module regulates transcriptional responses to diverse stress signals in bacteria. Mol Cell. 2007;27:793–805.

Supplementary Figure S1

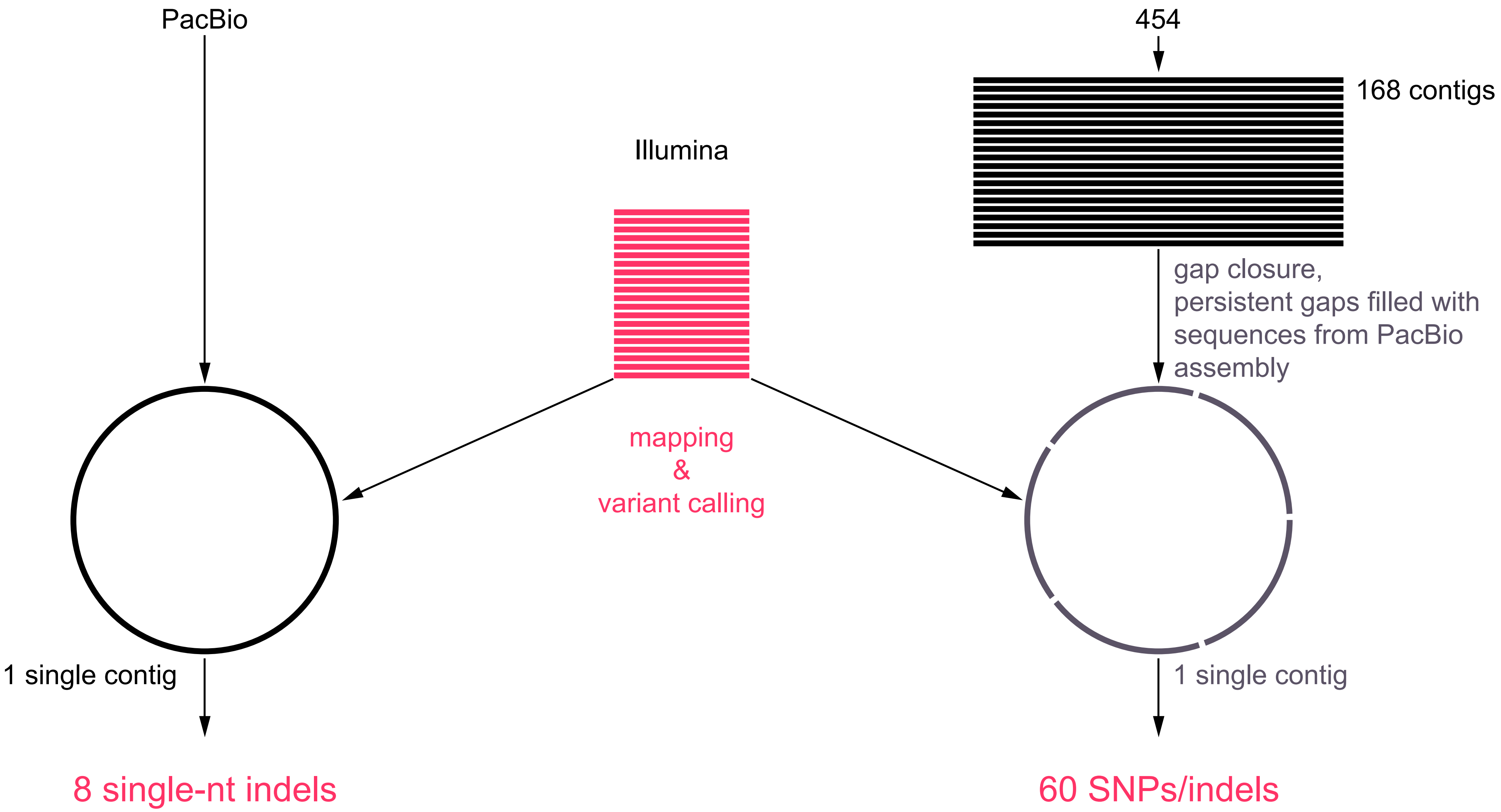

Supplementary Figure S2

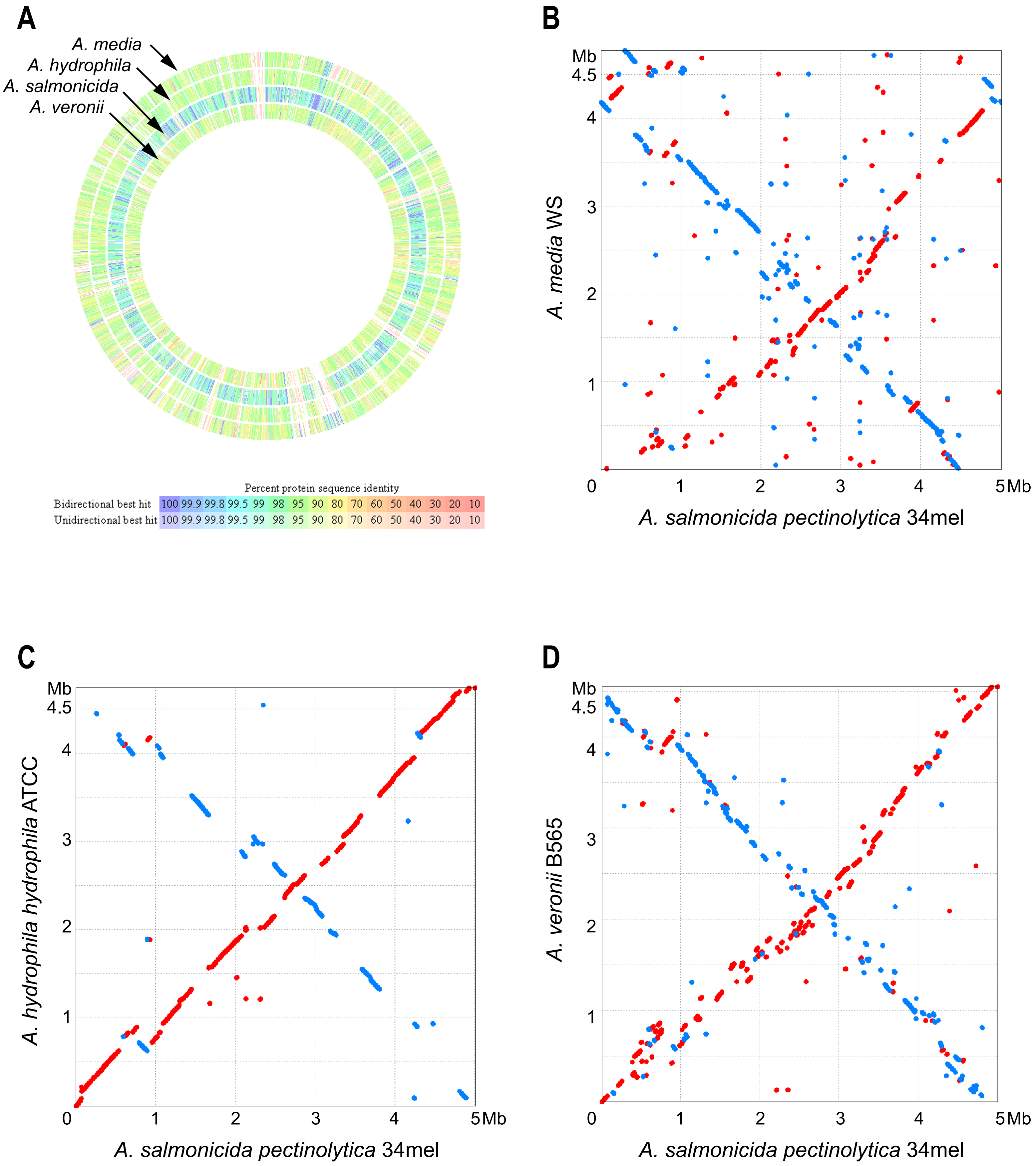

Supplementary Figure S3

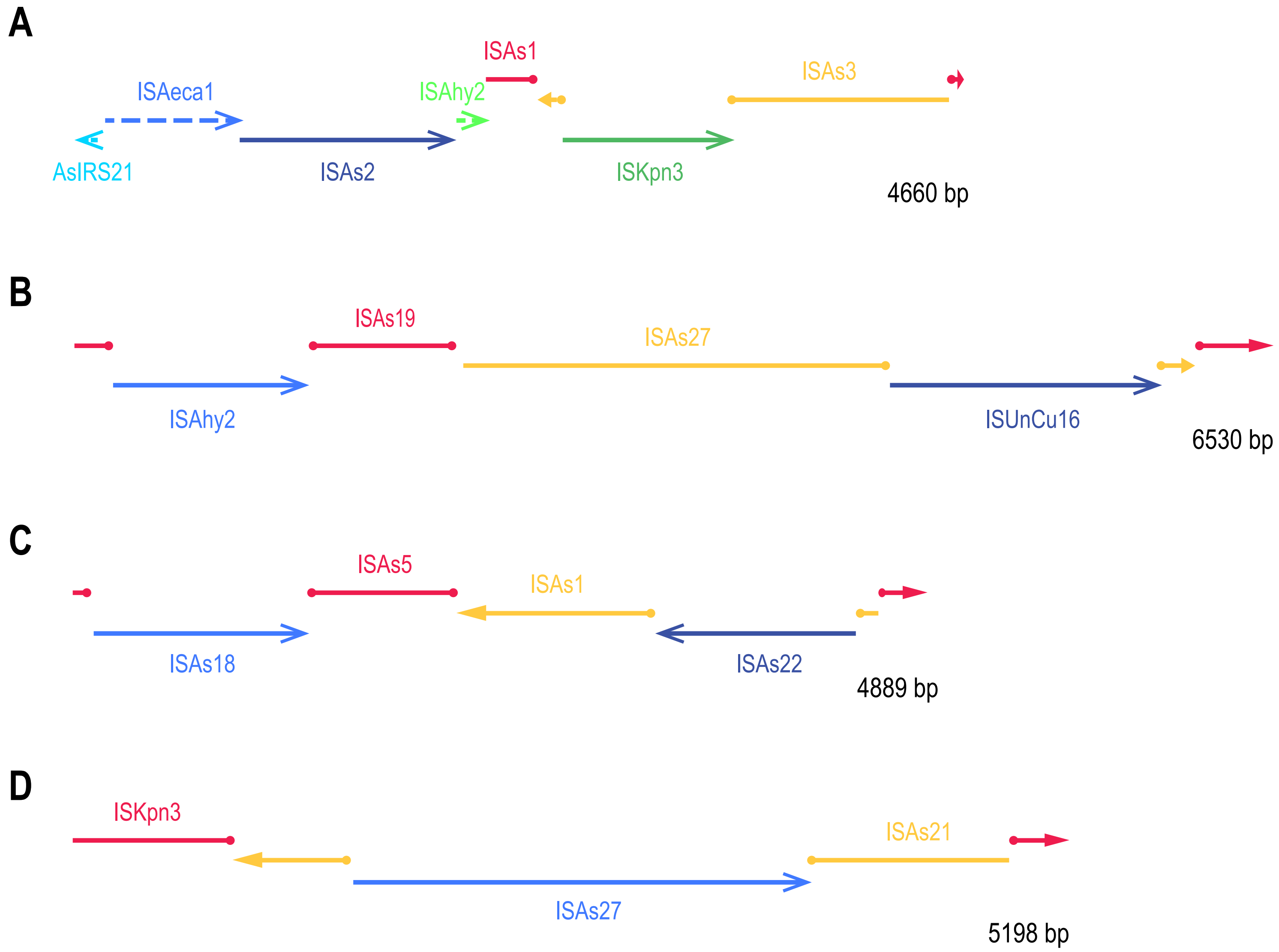

Supplementary Figure S4

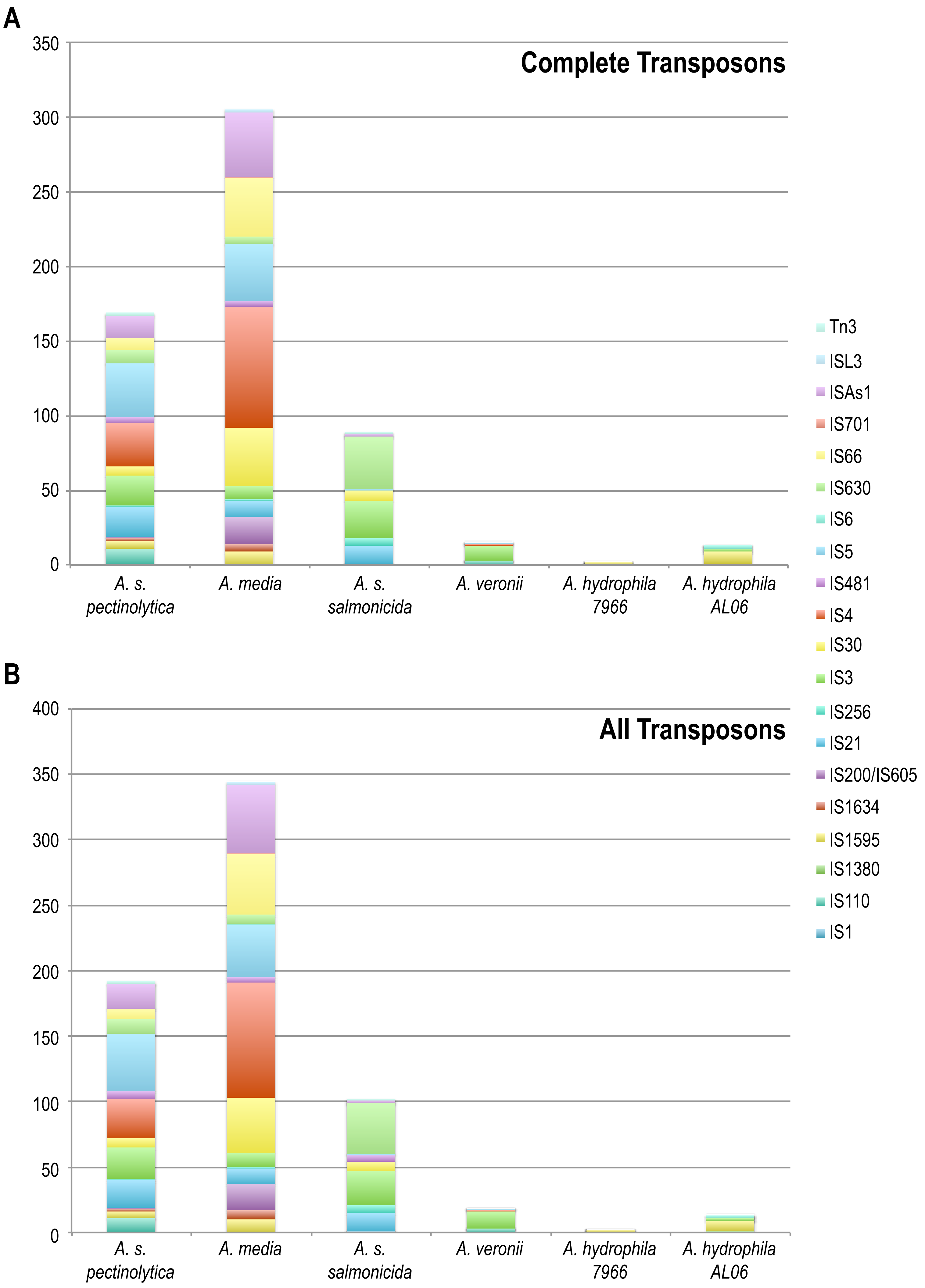

Supplementary Figure S5

A

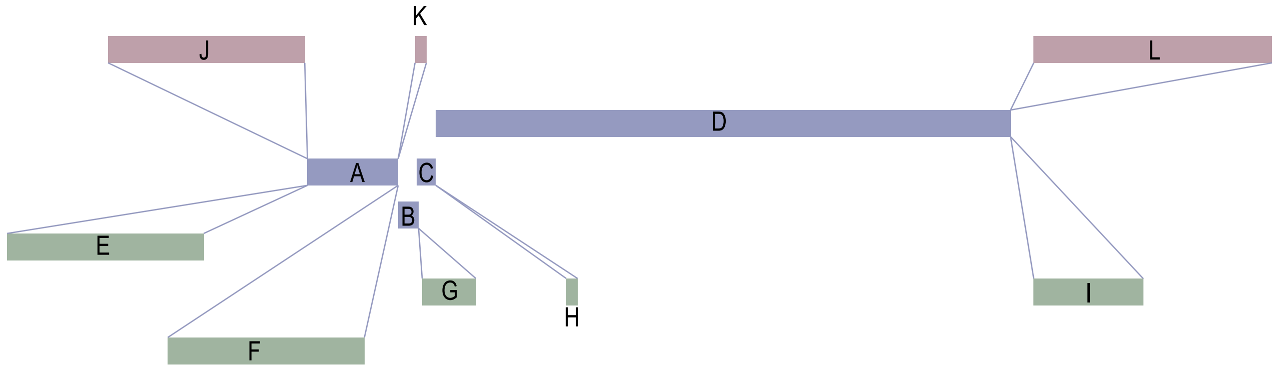

B

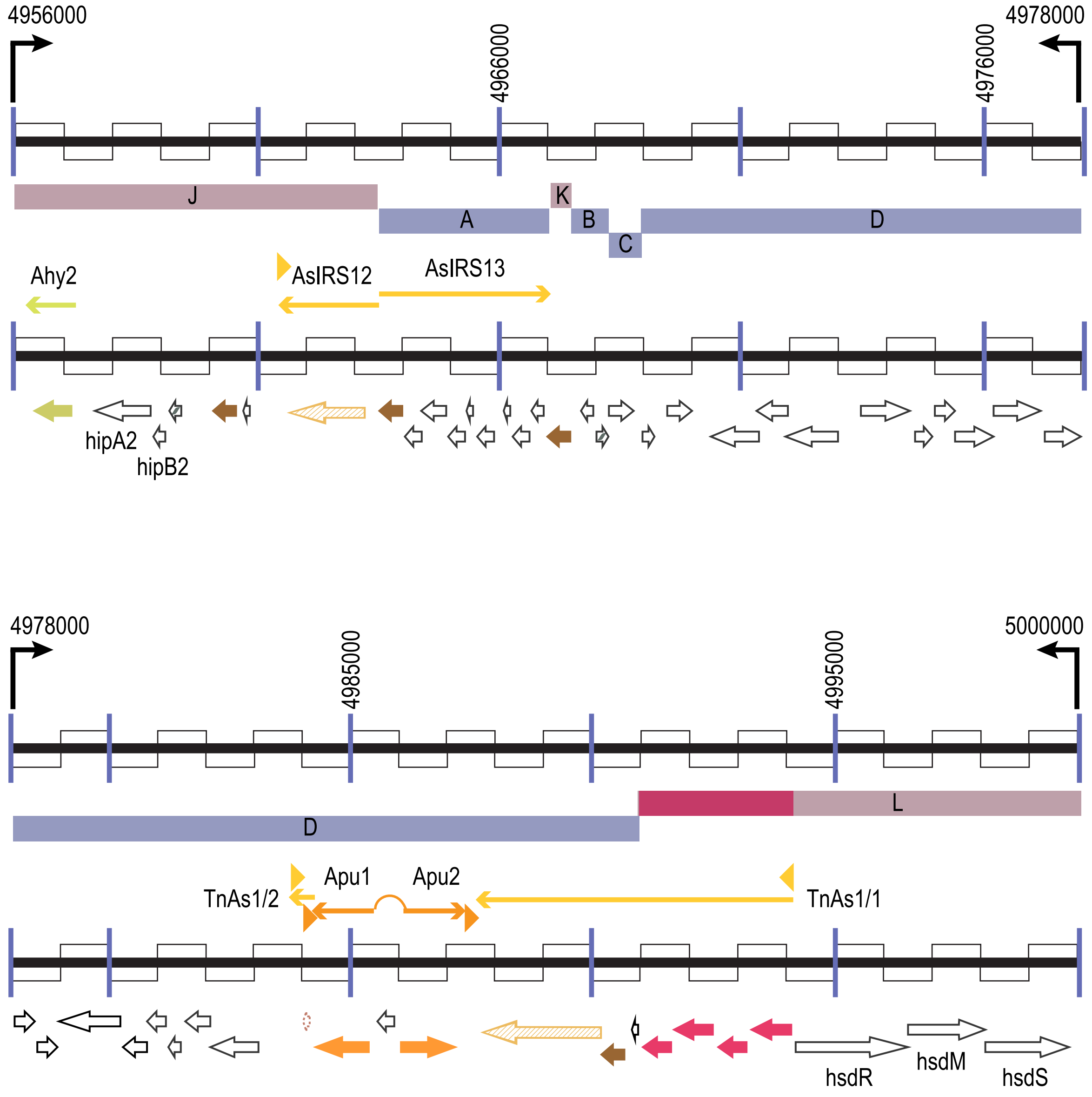

Supplementary Figure S5

C

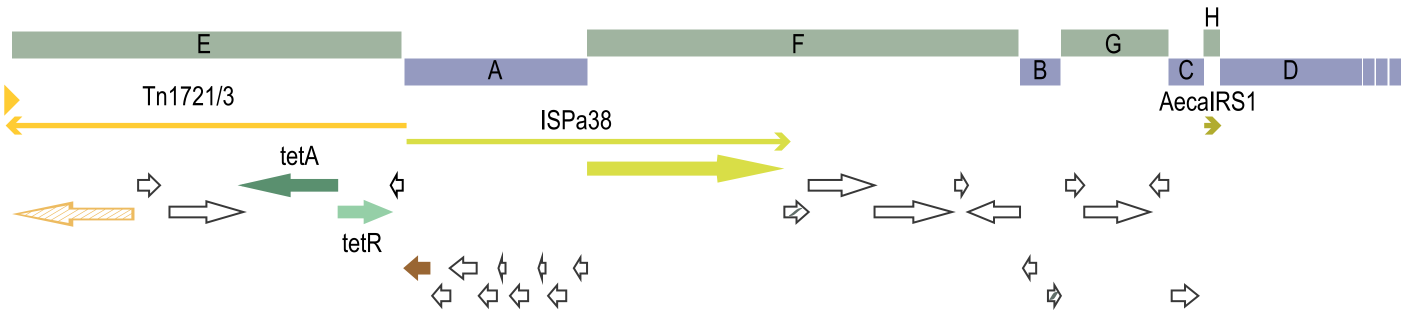

D

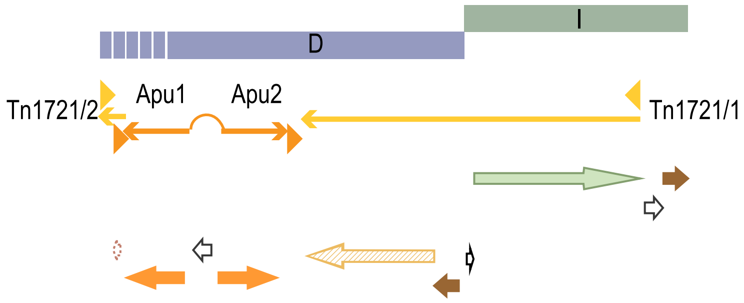

E

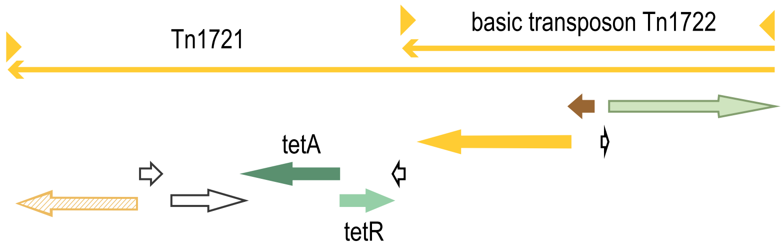

Supplement: Additional file 1: — Figure S1. Sequencing and assembly strategy of the A. salmonicida subsp. pectinolytica strain 34mel genome. Figure S2. Gene similarity and Mummer alignments to other Aeromonas genomes. Figure S3. Transposon conglomerates from A. salmonicida subsp. pectinolytica strain 34mel. Figure S4. Transposon family content in analyzed Aeromonas strains. Figure S5. Comparison of the A. salmonicida subsp. pectinolytica strain 34mel genome, plasmid pFBAOT6 from Aeromonas caviae, and transposon Tn1721. Table S1. Pathogenic as well as temperature-dependent growth features of analyzed Aeromonas strains. Table S2. General genome features of A. salmonicida subsp. pectinolytica strain 34mel. Table S3. Polymorphic sites in rRNAs from A. salmonicida subsp. pectinolytica strain 34mel. Table S4. Genes disrupted by transposons in A. salmonicida subsp. pectinolytica strain 34mel. Table S5. Transposon details from analyzed Aeromonas strains. Table S6. Genes retained in transposon TnAs1 from the environmental IncP-9 TOL plasmid pWW0 from Pseudomonas putida. Text S1. Annotation details for RNA genes in the A. salmonicida subsp. pectinolytica strain 34mel genome. Text S2. Short unassigned contigs from the A. salmonicida subsp. pectinolytica strain 34mel draft genome. Text S3. Transposons: nomenclature and completeness. Text S4. On transposon counting and the meaning of “ISAs11”. Text S5. Insertions on pFBAOT6 in transposon Tn1721 and details of the regions shared with the A. salmonicida subsp. pectinolytica strain 34mel genome. Text S6. Details of PacBio library preparation, sequencing, and assembly of the genome from A. salmonicida subsp. pectinolytica strain 34mel. (PDF 1171 kb) [file 12864_2017_4301_MOESM1_ESM.pdf]
